# iPLA2β-dependent phospholipid remodeling drives ZIKV-induced neuroinflammation and neurological injury

**DOI:** 10.1186/s12974-026-03973-2

**Published:** 2026-07-20

**Authors:** Li Jiao, Wenxin Shao, Ying Xie, Sirong Li, Jiahong Gao, Jianyu Xu, Rui Tong, Dehong Li, Qiangming Sun

**Affiliations:** 1 Institute of Medical Biology, Chinese Academy of Medical Sciences, Peking Union Medical College, 935 Jiaoling Road, Kunming, Yunnan 650118 China; 2 Kunming Medical University, Kunming, 650500 People’s Republic of China; 3 State Key Laboratory of Respiratory Health and Multimorbidity, Beijing, China; 4 Key Laboratory of Pathogen Infection Prevention and Control (Peking Union Medical College), Ministry of Education, Beijing, China; 5 National Kunming High-Level Biosafety Primate Research Center, Institute of Medical Biology, Chinese Academy of Medical Sciences and Peking Union Medical College, Yunnan, China; 6 Yunnan Key Laboratory of Vaccine Research & Development on Severe Infectious Diseases, Kunming, Yunnan Province China; 7 Yunnan Key Laboratory of Vector-borne Infectious Disease, Kunming, Yunnan Province China

## Abstract

Zika virus (ZIKV) infection of the developing brain induces neuroinflammatory responses that can restrict viral replication but may also contribute to neurological injury when dysregulated. Although neuronal phospholipid homeostasis is critical for membrane integrity and synaptic function, how ZIKV infection reprograms lipid metabolism to drive neuropathogenesis remains unclear. Here, we identify calcium-independent phospholipase A2β (iPLA2β) as a key mediator of ZIKV-induced neuronal lipid remodeling. iPLA2β is rapidly upregulated in neurons and in the neonatal brain following infection, and its expression is promoted by the viral envelope protein, which interacts with iPLA2β in neurons. Genetic ablation of iPLA2β reduces viral burden in the developing brain and in primary neurons and disrupts ZIKV-induced phospholipid remodeling, characterized by altered arachidonic acid–containing phospholipids and depletion of docosahexaenoic acid-enriched species. Importantly, sustained iPLA2β activity promotes phospholipid remodeling that supports efficient ZIKV replication, thereby increasing viral burden and contributing to a pro-inflammatory lipid environment. This is associated with increased prostaglandin E₂ production, glial activation, and neuronal loss. Both genetic and pharmacological inhibition of iPLA2β partially restores phospholipid homeostasis and reduces viral burden, accompanied by attenuation of neuroinflammatory responses. Functionally, targeting iPLA2β improves survival and neurobehavioral outcomes and mitigates long-term cognitive deficits following neonatal ZIKV infection. Collectively, these findings support iPLA2β-dependent phospholipid remodeling as a host process that facilitates ZIKV replication and links infection to neuroinflammation and neurological injury, highlighting iPLA2β as a potential therapeutic target in ZIKV-associated neuropathogenesis.

**Supplementary Information:**

The online version contains supplementary material available at https://doi.org/10.1186/s12974-026-03973-2.

## Introduction

Zika virus (ZIKV) is an emerging mosquito-borne flavivirus with strong neurotropism and the potential to cause severe neurological disorders [1]. Significant outbreaks in the Americas during 2015–2016 established a causal relationship between maternal infection and congenital microcephaly [2]. In addition to congenital infection, ZIKV affects postnatal and adult brains, targeting neural progenitor cells, glial cells, and mature neurons [3], leading to neuronal impairment and disrupted neural circuitry [4, 5]. Neuroinflammation is a hallmark of ZIKV infection, characterized by microglial activation, astrocyte reactivity, and elevated pro-inflammatory cytokine levels [6], and is closely associated with neuronal injury and long-term neurological deficits. Clinical evidence further indicates that even in the absence of congenital microcephaly, prenatal exposure to ZIKV is associated with postnatal neurodevelopmental abnormalities [7, 8], accompanied by neuronal loss and inflammatory pathology.

As essential components of biological membranes, phospholipids not only maintain membrane structural stability but also participate in lipid-mediated signaling pathways [9]. Neurons highly depend on phospholipid metabolism to preserve membrane integrity, support synaptic transmission, and regulate intracellular signal transduction. Enveloped viruses, including ZIKV, can hijack host membrane systems to facilitate key stages of their life cycle, including cell entry, genome replication, and virion assembly [10, 11]. These processes require coordinated remodeling of membrane lipid composition and biophysical properties [12]. Such lipid remodeling facilitates viral replication, which in turn contributes to cellular stress and inflammatory signaling [11, 13]. During ZIKV infection, membrane remodeling facilitates envelope–endosomal fusion through changes in phospholipid organization [14] and is accompanied by the accumulation of glycerophospholipids in infected neural cells, which has been linked to enhanced viral replication and virion formation [15].

Phospholipid metabolism consists of de novo synthesis and remodeling pathways. Compared with de novo synthesis, phospholipid remodeling generates structurally diverse lipid species through phospholipase-mediated deacylation, followed by reacylation by lysophospholipid acyltransferases [16]. This remodeling process is essential for vesicular trafficking that supports viral replication. In dengue virus infection, phospholipid remodeling is activated early and promotes lipid diversity and membrane adaptation, supporting efficient viral replication [17]. However, the phospholipase systems responsible for regulating phospholipid remodeling in ZIKV-infected neurons remain unclear.

Among phospholipid remodeling enzymes, phospholipase A2 (PLA2) hydrolyzes glycerophospholipids to generate free fatty acids and lysophospholipids (LPLs). iPLA2β (PLA2G6), a major neuronal PLA2 isoform, regulates phospholipid homeostasis by controlling the membrane lipid composition and fatty acid metabolism [18, 19]. iPLA2β dysfunction has been implicated in neuroaxonal dystrophy and dystonia-Parkinsonism, suggesting its involvement in the maintenance of neuronal lipid homeostasis [9, 19]. Notably, iPLA2β-mediated phospholipid hydrolysis leads to the release of arachidonic acid (AA), thereby linking membrane remodeling to inflammatory lipid signaling cascades [20]. AA is a precursor of eicosanoid synthesis, including prostaglandin E2 (PGE_2_), which is a key mediator of neuroinflammatory response. Elevated PGE_2_ levels promote cytokine production and neuronal injury in viral infection models [21] and are also increased during ZIKV infection, contributing to neuroinflammatory damage [22], which may be further amplified by enhanced viral replication.

In this study, we hypothesized that ZIKV infection alters neuronal phospholipid metabolism through iPLA2β, thereby supporting viral replication and contributing to downstream neuroinflammatory responses and neuronal injury. Using neonatal mouse infection models and primary neuronal systems, we observed that ZIKV infection reshaped the neuronal phospholipid composition, characterized by increased arachidonic acid-containing species and decreased DHA-containing species. This remodeling was accompanied by the upregulation of iPLA2β driven by the viral envelope (E) protein. Notably, both genetic deletion and pharmacological inhibition of iPLA2β attenuated ZIKV replication, reduced phospholipid remodeling, and alleviated neuroinflammatory responses and neuronal injuries. These findings identify iPLA2β-dependent phospholipid remodeling as a key pathway that facilitates ZIKV replication and links infection to downstream neuroinflammatory responses and neuronal injury.

## Materials and methods

### Viral propagation and titration

The clinical isolate ZIKV SZ01 was propagated in Vero cells (ATCC CCL-81). Briefly, cells were cultured in DMEM supplemented with 10% fetal bovine serum and 100 U/mL penicillin–streptomycin. Virus-containing supernatants were collected once extensive cytopathic changes were observed, followed by clarification through centrifugation (3,000 × g, 10 min). After aliquoting, viral stocks were stored at − 80 °C. Virus titers were measured by standard plaque assay using Vero cell monolayers and expressed as PFU/mL.

### Animals

Animal experimentation was conducted with the permission of Institutional Ethics Committee of the Chinese Academy of Medical Sciences and Peking Union Medical College in accordance with the appropriate rules of animal welfare laboratories. The iPLA2β⁻/⁻ mice were purchased from Shanghai Model Organisms Center (Shanghai, China). All animals were maintained on a C57BL/6 background and were kept in specific pathogen-free conditions at the Institute of Biology, Chinese Academy of Medical Sciences. The neonatal mice of both sexes were randomly allocated to their respective experimental groups.

### ZIKV infection and in vivo drug administration

Neonatal C57BL/6 mice at postnatal day 1 (P1) were anesthetized by hypothermia on a chilled surface until loss of righting and withdrawal reflexes. Intracerebroventricular (i.c.v.) injections were performed using a Hamilton microsyringe fitted with a 33-gauge needle and a pulled glass capillary tip. The injection site was defined based on anatomical landmarks, approximately 1 mm lateral to the midline at the level between the eyes, targeting the lateral ventricle. A total volume of 1 µL of ZIKV suspension (2 × 10³ or 2 × 10⁴ PFU) or PBS was injected at a rate of 0.2–0.5 µL/min. Following injection, pups were placed on a 37 °C heating pad for recovery and returned to the dam after full recovery. Maternal acceptance was ensured by briefly rubbing pups with nesting material. Injection accuracy was validated in pilot experiments using dye co-injection.

**Fig. 1:**
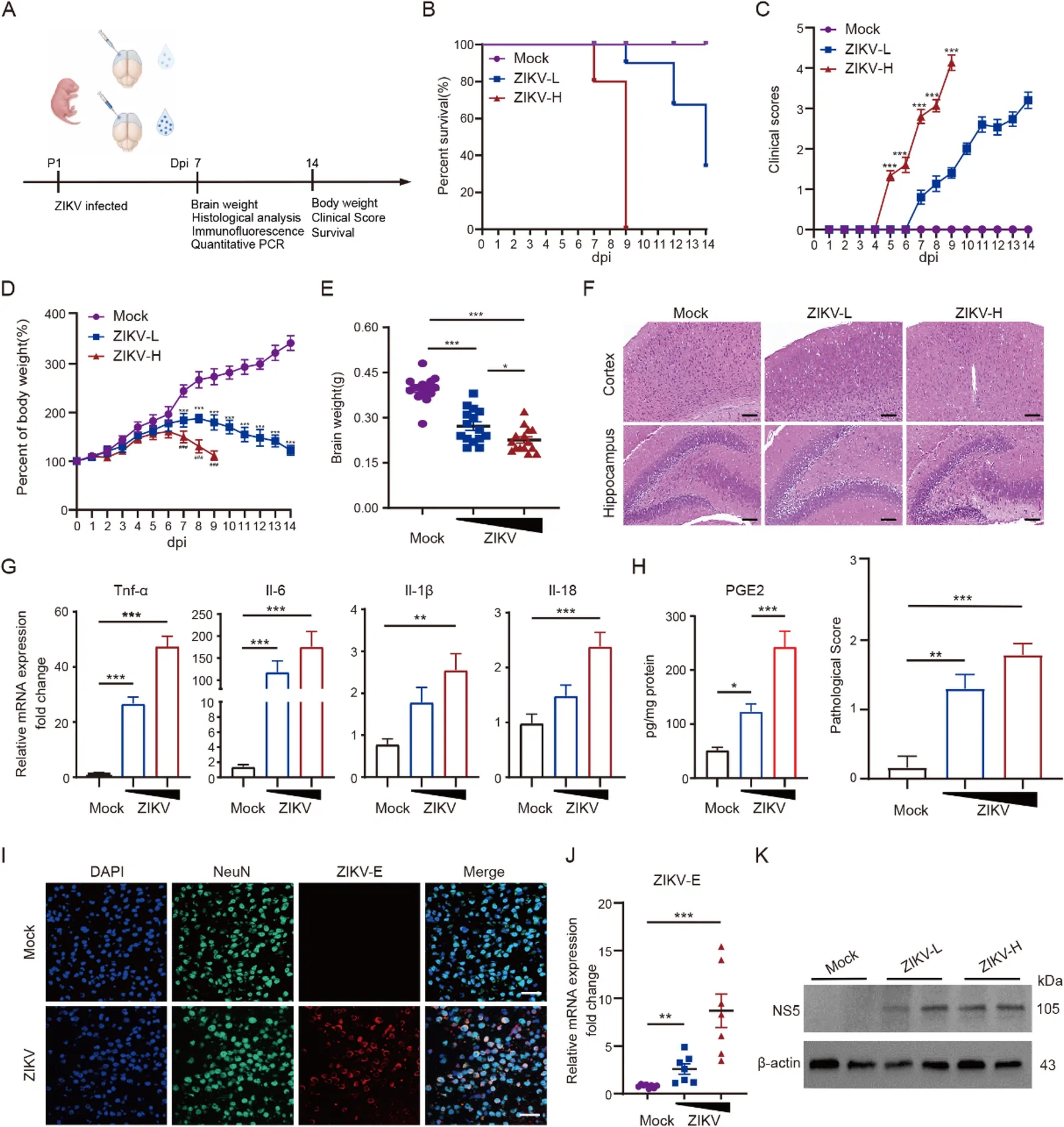
ZIKV infection induces neuroinflammation and neuronal infection in the developing brain. **A** Schematic illustration of the experimental design. Postnatal day 1 (P1) C57BL/6 mice received intracerebroventricular injections of either a low dose (10^4^ PFU) or a high dose (10^5^ PFU) of the ZIKV SZ01 strain. **B** Survival curves of control and ZIKV-infected neonatal mice. *n* = 10. **C** Clinical scores of mice following ZIKV infection. *n* = 10. **D** Changes in body weight of control and ZIKV-infected mice during infection. *n* = 10. **E** Brain weights of mice at 7 dpi. *n* = 15. **F** Representative H&E-stained images of the cortex and hippocampus at 7 dpi. Scale bar, 100 μm. n = 6. **G** Relative mRNA expression levels of pro-inflammatory cytokines in the cortices at 7 dpi, determined by quantitative real-time PCR (qPCR). *n* = 6. **H** Prostaglandin E₂ (PGE₂) levels in the cerebral cortex at 7 dpi. *n* = 6. **I** Representative immunofluorescence images showing ZIKV E (red) and NeuN (green) in the cerebral cortex at 7 dpi. Scale bars, 50 μm. **J** Relative ZIKV E mRNA expression levels in primary mouse cortical neurons 48 h post-infection, as determined by qPCR. n = 7. **K** Representative western blot analysis of NS5 protein expression in primary mouse cortical neurons at 48 h post-infection. Data are presented as mean ± SEM. Statistical significance was determined using the Mann–Whitney U test for (C) and one-way ANOVA followed by post hoc Tukey’s multiple comparison test (D–J). *p < 0.05, **p < 0.01, ***p < 0.001

For the dose-response study shown in Fig. 1, neonatal mice were infected with either 2 × 10³ or 2 × 10⁴ PFU ZIKV. Based on these results, all subsequent mechanistic experiments (Figs. 3, 4, 5 and 6) were performed using the 2 × 10⁴ PFU i.c.v. infection model. A separate cohort of infected mice was monitored daily for survival for 14 days post-infection. Clinical status was evaluated using a previously established scoring system [23], including rough fur (1 point), prominent bones (2 points), hunched posture (2 points), reduced activity (2 points), tremor (3 points), and seizures (4 points). For long-term experiments (Figs. 7), mice were intraperitoneally inoculated with 2 × 10⁵ PFU ZIKV, while littermate controls received an equal volume of PBS. The ZIKV strain source and viral titers were determined by standard plaque assay. FKGK18 (15 mg/kg; MedChem Express, HY-115403) or 18:0–22:6 phosphatidylethanolamine (PE) (10 mg/kg; MedChem Express, HY-157624) was administered intraperitoneally on days 0 and 3 post-infection, with corresponding vehicle controls (DMSO for FKGK18 and methanol for PE).

### Cell culture and in vitro ZIKV infection

SH-SY5Y cells (ATCC CRL-2266) were propagated in DMEM supplemented with 10% fetal bovine serum and penicillin–streptomycin (100 U/mL). Cultures were maintained at 37 °C in a 5% CO₂ atmosphere. For infection studies, cells were challenged with ZIKV at an MOI of 1.0 and processed 48 h later for further analyses. Primary cortical neurons were generated from E17 C57BL/6 mouse embryos according to a previously described protocol [24]. Following cortical dissection, cells were dissociated and seeded in DMEM/F-12 containing 10% FBS. Three hours after plating, the medium was exchanged for Neurobasal medium supplemented with B27, 2 mM L-glutamine, penicillin (0.1 U/mL), and streptomycin (0.1 µg/mL). Neuronal cultures were allowed to mature for 7 days before ZIKV exposure. FKGK18 (0.1 µM) or 18:0–22:6 phosphatidylethanolamine (50 µM) was administered at the onset of infection where indicated.

### Plasmid construction and cell transfection

The coding sequences corresponding to ZIKV structural and nonstructural proteins, including capsid (amino acids 6–122), envelope (302–749), NS1 (795–1148), NS2A (1158–1307), NS2B (1377–1502), NS3 (1521–1975), NS4A (2125–2269), NS4B (2270–2512), and NS5 (2774–3412), were amplified from the ZIKV SZ01 strain (GenBank: KU866423.2) and cloned into pcDNA3.1 expression vectors containing C-terminal Myc or Flag tags (Invitrogen, Carlsbad, CA, USA). SH-SY5Y cells (ATCC CRL-2266) were seeded in culture plates and transfected with 0.5–1 µg plasmid DNA using the Lipofectamine 3000 transfection reagent (Invitrogen, L3000015). Cells were harvested 48 h post-transfection for subsequent analysis. The expression of Myc-tagged ZIKV proteins and endogenous iPLA2β was evaluated by western blotting using the indicated antibodies.

### Histology and immunohistochemistry

Mice were anesthetized to a surgical plane by intraperitoneal injection of pentobarbital sodium (50 mg/kg) and transcardially perfused with phosphate-buffered saline (PBS), followed by 4% paraformaldehyde (PFA). Brains were post-fixed in 4% PFA at 4 °C overnight before paraffin processing. Fixed brains were sequentially dehydrated in graded ethanol solutions (70%, 80%, 95%, and 100%), cleared in xylene, infiltrated with molten paraffin, and embedded in paraffin blocks. Paraffin blocks were sectioned at a thickness of 5 μm using a rotary microtome (PM-24, Servicebio, Wuhan, China), and sections were mounted onto glass slides for subsequent histological and immunohistochemical analyses. For immunohistochemistry, sections were deparaffinized in xylene and rehydrated through graded ethanol solutions. Endogenous peroxidase activity was blocked with 3% hydrogen peroxide for 30 min. Antigen retrieval was performed in sodium citrate buffer (10 mM, pH 6.0) at 95–100 °C for 30 min. Sections were blocked with 5% bovine serum albumin (BSA) for 1 h at room temperature and incubated overnight at 4 °C with anti-iPLA2β antibody (1:100; Santa Cruz Biotechnology, sc-376563). After washing, sections were incubated with an HRP-conjugated secondary antibody for 1 h at room temperature. Immunoreactivity was visualized using 3,3′-diaminobenzidine (DAB, 0.02%) and counterstained with hematoxylin. Histological and immunohistochemical images were acquired using a bright-field microscope (SWE-CX63, Servicebio, Wuhan, China) equipped with a digital imaging system. Images were captured using Saiviewer software (Servicebio, Wuhan, China) under identical illumination, exposure time, and gain settings for all experimental groups. Representative images were obtained from anatomically matched brain regions. Histopathological scoring was performed in a blinded manner using a semi-quantitative scale (0–4) according to the INHAND guidelines [25].

### Quantitative real-time PCR (qRT-PCR)

TRIzol reagent (Invitrogen, USA) was used to extract total RNA from primary neurons and mouse tissues according to standard procedures. Viral RNA from supernatants was purified using a QIAamp Viral RNA Mini Kit (Qiagen, Germany). cDNA was generated from 1 µg of RNA using a reverse transcription kit (TaKaRa, Japan). Real-time PCR was conducted with SYBR Green chemistry (Promega, USA) using an initial denaturation step at 95 °C for 30 s, followed by 40 amplification cycles (95 °C for 10 s and 60 °C for 30 s). Product specificity was verified by melt curve analysis. For viral load determination, absolute quantification was performed using plasmid-based standard curves generated from serial 10-fold dilutions. Relative expression of target genes was normalized to Gapdh using the 2^-ΔΔCt method. All reactions were performed in technical triplicate. The primer sequences used in this study are listed below: ZIKV E (NC_012532.1)-forward: 5′- GACCGGGAAGAGCATTCAAC − 3′, -reverse: 5′- CCGCTCTTGGTGAATTAGGC − 3′; mouse Gapdh (NM_001289726.2)-forward: 5′- AACGGATTTGGCCGTATTGG-3′, -reverse: 5′- CATTCTCGGCCTTGACTGTG-3′; mouse Pla2g6 (NM_016915)-forward: 5′- GCAAGATGGGAAAGCAGGAG-3′, -reverse: 5′- CTTCATGGCTGTGTGGATGG-3′; mouse Il-18 (NM_008360.2) -forward: 5′- GCTGTGACCCTCTCTGTGAA-3′, -reverse: 5′- TGGCAAGCAAGAAAGTGTCC-3′; mouse Tnf (NM_013693.3) -forward: 5′- CCAACGGCATGGATCTCAAA-3′, - reverse: 5′- CCCTTGAAGAGAACCTGGGA-3′; mouse Il-1β (NM_008361.4) -forward: 5′- ACTCATTGTGGCTGTGGAGA-3′, -reverse: 5′- AGCCTGTAGTGCAGTTGTCT-3′; mouse Il-6 (NM_031168.2) -forward: 5′-ACTTCACAAGTCCGGAGAGG-3′,-reverse: 5′- TGCAAGTGCATCATCGTTGT-3′; mouse Plb1 (NM_001081407.1) -forward: 5′- TATGAGCTGGCAAGGTTGGA-3′, -reverse: 5′- GAAGTCCTCTCGAGTGTCGT-3′; mouse Pla2g2a (NM_001082531.1) -forward: 5′- ACAAGTACTCCCACCAAGGG-3′, - reverse: 5′- CGGCTTTATCGCACTGACAC-3′; mouse Pla2g4a (NM_008869.4) -forward: 5′- TTGCCTCTCTTCACGTGTCT-3′, - reverse: 5′- ATTCCACCCAATCGGCAAAC-3′; mouse Chka (NM_013490.5) -forward: 5′- CAGAGTTCAGCAACTGCACA-3′, - reverse: 5′- TCTCCTGGCCTTCCAACAAT-3′; mouse Dgkd (NM_177646.3)-forward: 5′- CATGCAGATGGCTGTGTCTC-3′, -reverse: 5′- GTTCTTGTGCACGATCCGAA-3′。.

### Western blot analysis

Cell and tissue homogenates were lysed in ice-cold RIPA buffer containing protease and phosphatase inhibitors (Proteintech, USA) on ice for 20 min. Lysates were clarified by centrifugation at 12,000 × g for 15 min at 4 °C, and the resulting supernatants were collected for protein quantification using a BCA assay kit (Proteintech, USA). Equal amounts of protein (30 µg) were resolved by 10% SDS-PAGE and transferred onto 0.45 μm PVDF membranes. Membranes were blocked with 5% non-fat milk for 1 h at room temperature and subsequently incubated with primary antibodies at 4 °C overnight. After washing three times, membranes were incubated with HRP-conjugated secondary antibodies (1:5000) for 1 h at room temperature. Protein bands were developed using ECL reagents (Proteintech, USA). Primary antibodies used included anti-ZIKV NS5 (1:1000, GeneTex, GTX133312), anti-ZIKV E protein (1:1000, GeneTex, GTX133314), anti-iPLA2β (1:1000, Santa Cruz, sc-376563), anti-Myc (1:1000, CST, #5605), anti-cleaved caspase-3 (1:1000, CST, #9661), and anti-β-actin (1:5000, Proteintech, USA).

### Co-immunoprecipitation (Co-IP)

Protein extraction and quantification were performed as described in the Western blot analysis section. Immunoprecipitation was performed using an IP kit (Beyotime, China) according to the manufacturer’s instructions. Briefly, 1 mg of total protein lysate was incubated with anti-Myc antibody (10 µg) at 4 °C overnight, followed by incubation with Protein A/G magnetic beads at 4 °C for 4 h. Immunocomplexes were washed three times and eluted in SDS loading buffer for Western blot analysis, with input as control.

### Prostaglandin E₂ quantification

Brain tissues and primary neuronal cells were lysed in PBS buffer with 1 mM PMSF. Prostaglandin E₂ (PGE₂) levels were measured using a commercial ELISA kit (FineTest, EM1503, China) according to the manufacturer’s instructions. Briefly, brain tissues and primary neuronal cells were lysed in PBS containing 1 mM PMSF, and protein concentrations were determined using a BCA assay. Serial dilutions of standards were prepared at 1000, 500, 250, 125, 62.5, 31.25, and 0 pg/mL. 50 µL of standards or samples were added to a 96-well plate, followed by 50 µL of biotin-labeled antibody working solution. After incubation at 37 °C for 45 min with gentle shaking, the plate was washed thoroughly, and 100 µL of HRP–SABC solution was added for further incubation at 37 °C for 30 min. After washing, 90 µL of TMB substrate solution was added for color development in the dark at 37 °C for 15 min. The reaction was terminated by adding 50 µL of stop solution, and absorbance was measured at 450 nm. PGE₂ concentrations were calculated using a standard curve generated from the corresponding standards.

### Plasma membrane fluidity assay

Membrane dynamics were assessed using the fluorescent probe TMA-DPH (N, N, N-trimethyl-4-(6-phenyl-1,3,5-hexatrien-1-yl) phenylammonium p-toluenesulfonate), which incorporates into the lipid bilayer of the plasma membrane. SH-SY5Y cells were seeded into 96-well plates at approximately 5 × 10³ cells per well. Cells were incubated with 5 µM TMA-DPH prepared in anhydrous tetrahydrofuran (THF) for 15 min in the dark at 37 °C. Following incubation, excess probe was removed by two washes with pre-warmed PBS. Parallel wells received vehicle containing equivalent THF concentration as controls. Fluorescence polarization was measured using excitation and emission wavelengths of 355 nm and 430 nm, respectively. Emission intensities were recorded using vertical (I_V) and horizontal (I_H) analyzer orientations under vertically polarized excitation. Membrane fluidity was inferred from fluorescence anisotropy (r), calculated according to the standard polarization equation with instrumental correction factor G:

$$r=\frac{I_v-GI_H}{I_v+2GI_H}$$

Increased anisotropy corresponds to reduced membrane fluidity.

### Ca²⁺-independent phospholipase A2 activity assay

iPLA2 enzymatic activity in primary cortical neurons was measured using a commercial PLA2 activity assay kit (Abcam, ab133089, UK) with minor modifications [26]. Briefly, cell lysates were prepared in ice-cold assay buffer, and protein concentrations were determined using a BCA assay. Equal amounts of protein were used for each reaction under Ca²⁺-free conditions, and EGTA was included to selectively suppress Ca²⁺-dependent PLA2 isoforms. Reactions were set up in a 96-well plate including blank, positive control, and sample wells. Blank wells contained DTNB and assay buffer; positive control wells contained DTNB, bee venom PLA2, and assay buffer; sample wells contained DTNB and samples (or inhibitors where applicable). All reactions were initiated by adding 200 µL substrate solution and gently mixing by plate shaking. Absorbance was recorded at 414 nm at 1-min intervals for five time points using a microplate reader. Enzymatic activity was calculated based on the linear rate of absorbance change over time (ΔA/min).

### RNA sequencing and bioinformatic analysis

TRIzol-extracted RNA from mouse cortex and primary neurons was used for transcriptomic profiling after quality assessment. Poly(A)-selected, strand-specific libraries were generated and sequenced on an Illumina NovaSeq 6000 platform (150 bp paired-end reads) by BGI Metabolism (China). Sequencing reads were processed using fastp to remove adapters, low-quality bases (Phred < 20), and short reads (< 30 bp). Clean reads were mapped to the mouse genome (GRCm38/mm10) using STAR, and uniquely aligned reads were retained. Gene expression quantification was performed with featureCounts. Differential gene expression analysis was conducted using DESeq2. Transcripts with |log₂FC| ≥ 0.3 and adjusted *P* < 0.05 (Benjamini–Hochberg) were considered significant DEGs. Pathway and functional enrichment analyses were performed using IPA (QIAGEN, Germany) under default settings.

### Untargeted lipidomics analysis by LC–MS/MS

Approximately 10 mg of mouse brain tissue was subjected to MTBE-based lipid extraction using a modified protocol described previously. Samples were homogenized in 400 µL ultrapure water and mixed with extraction solvents (Biotree, Shanghai, China). Following vortexing and incubation, lipid phases were separated by centrifugation. Additional MTBE was introduced, and after sonication and a second centrifugation step, the upper organic layer was recovered. Collected lipid fractions were vacuum-dried and reconstituted in solvent prior to LC–MS analysis. Lipid separation was performed on an Agilent 1290 UHPLC system (Agilent Technologies, USA) using an ACQUITY UPLC HSS T3 column (Waters, USA). Lipid profiling and identification were carried out using Biobud v2.0.7 software (Biotree, China) according to standard operating procedures.

### Behavioral tests

#### Morris water maze

Morris water maze was used to assess spatial learning and memory as previously described [27]. A circular pool was filled to a depth of approximately 30 cm, and a non-toxic opacifying agent was added to the water to facilitate tracking of dark-coated mice. The water temperature was maintained at 23–25 °C. The pool was divided into four equal quadrants (northeast, northwest, southeast, and southwest). A circular escape platform (10 cm in diameter) was placed in the center of the northeast quadrant and positioned approximately 2 cm below the water surface during the training phase, which lasted for 5 consecutive days. On day 6, the platform was removed, and mice were allowed to swim freely for 60 s during the probe trial. All trials were recorded using an overhead camera system and analyzed using ANY-maze software (SANS Biotechnology, China). Behavioral analysis was performed in a blinded manner.

### Rotarod test

Motor coordination and balance were tested using an accelerating rotarod. Mice were subjected to three trials (180 s each), during which rod speed increased from 5 to 20 rpm. Latency to fall was recorded automatically and verified by two blinded observers. Animals remaining on the rod for the full duration were scored as 180 s. The mean value across trials was used for analysis.

### Elevated plus maze (EPM)

Anxiety-like behavior was evaluated using the elevated plus maze (EPM). The apparatus was elevated 50 cm above the floor and consisted of two open arms and two closed arms arranged in a plus-shaped configuration around a central platform. Each mouse was placed in the center of the maze facing an open arm and allowed to explore freely for 10 min. All behavioral sessions were recorded using an overhead video tracking system. Time spent in each arm and the number of arm entries were quantified using ANY-maze software. All analyses were performed under blinded conditions.

### Novel object recognition (NOR) test

Recognition memory was assessed using a novel object recognition task in an open-field arena. Prior to testing, mice were habituated to the empty arena for 10 min per day. During the training phase, mice were exposed to two identical yellow wooden cube-shaped objects (8 cm × 8 cm × 8 cm) and allowed to explore freely for 5 min. After a 24 h retention interval, one familiar object was replaced with a novel yellow wooden spherical object (6 cm in diameter), while the other familiar object remained unchanged. Mice were then allowed to freely explore the two objects for 5 min under identical experimental conditions. An exploration event was defined as sniffing or direct physical contact with the object. Behavioral activity was recorded and analyzed using ANY-maze software (Stoelting Co., Wood Dale, IL, USA). Recognition memory was quantified using the recognition index (RI = TN/(TN + TF)) and discrimination index (DI = (TN − TF)/(TN + TF)), where TN represents the time spent exploring the novel object and TF represents the time spent exploring the familiar object.

### Statistical analysis

Statistical analyses were carried out using GraphPad Prism 8. Data are presented as mean ± SEM. Normality of distribution was assessed using the Shapiro–Wilk test before further comparisons. For two-group comparisons, unpaired two-tailed Student’s t-tests were used when data were normally distributed, with Welch’s correction applied in cases of unequal variances. The Mann–Whitney U test was applied for non-parametric data. For multiple-group comparisons, one-way ANOVA followed by Tukey’s post hoc test was performed. Statistical significance was defined as *p* < 0.05, *p* < 0.01, and *p* < 0.001.

## Results

### ZIKV infection induces neuroinflammation and neuronal infection in the developing brain

To examine the effects of ZIKV on the developing central nervous system, a neonatal intracerebroventricular (i.c.v.) infection model was established. C57BL/6 mice at postnatal day 1 (P1) were injected with either 10⁴ or 10⁵ PFU of the ZIKV SZ01 strain (Fig. 1A). Infected animals showed increased mortality (Fig. 1B), higher clinical scores (Fig. 1C), and reduced body weight gain compared with controls (Fig. 1D). Brain weight was also decreased at 7 days post-infection (dpi) (Fig. 1E). Histological analysis revealed pathological changes in the cortex and hippocampus, including perivascular inflammatory infiltration, glial activation, and neuronal loss after infection (Fig. 1F). Pro-inflammatory cytokines, including *Tnf-α*, *Il-6*, *Il-1β*, and *Il-18*, were significantly elevated, accompanied by increased levels of the inflammatory lipid mediator PGE₂ (Fig. 1G–H). In vivo immunofluorescence analysis of brain tissues at 7 dpi revealed extensive colocalization of ZIKV E with NeuN-positive neurons (Fig. 1I). To further evaluate neuronal infection, primary mouse cortical neurons were isolated and infected in vitro with ZIKV. At 48 h post-infection, qPCR and western blot analyses demonstrated a significantly increased expression of ZIKV E mRNA (Fig. 1J) and NS5 proteins (Fig. 1K), indicating that neurons are the major target cell type for ZIKV infection.

### ZIKV infection induces transcriptomic and lipid remodeling associated with iPLA2β activation in neurons

**Fig. 2:**
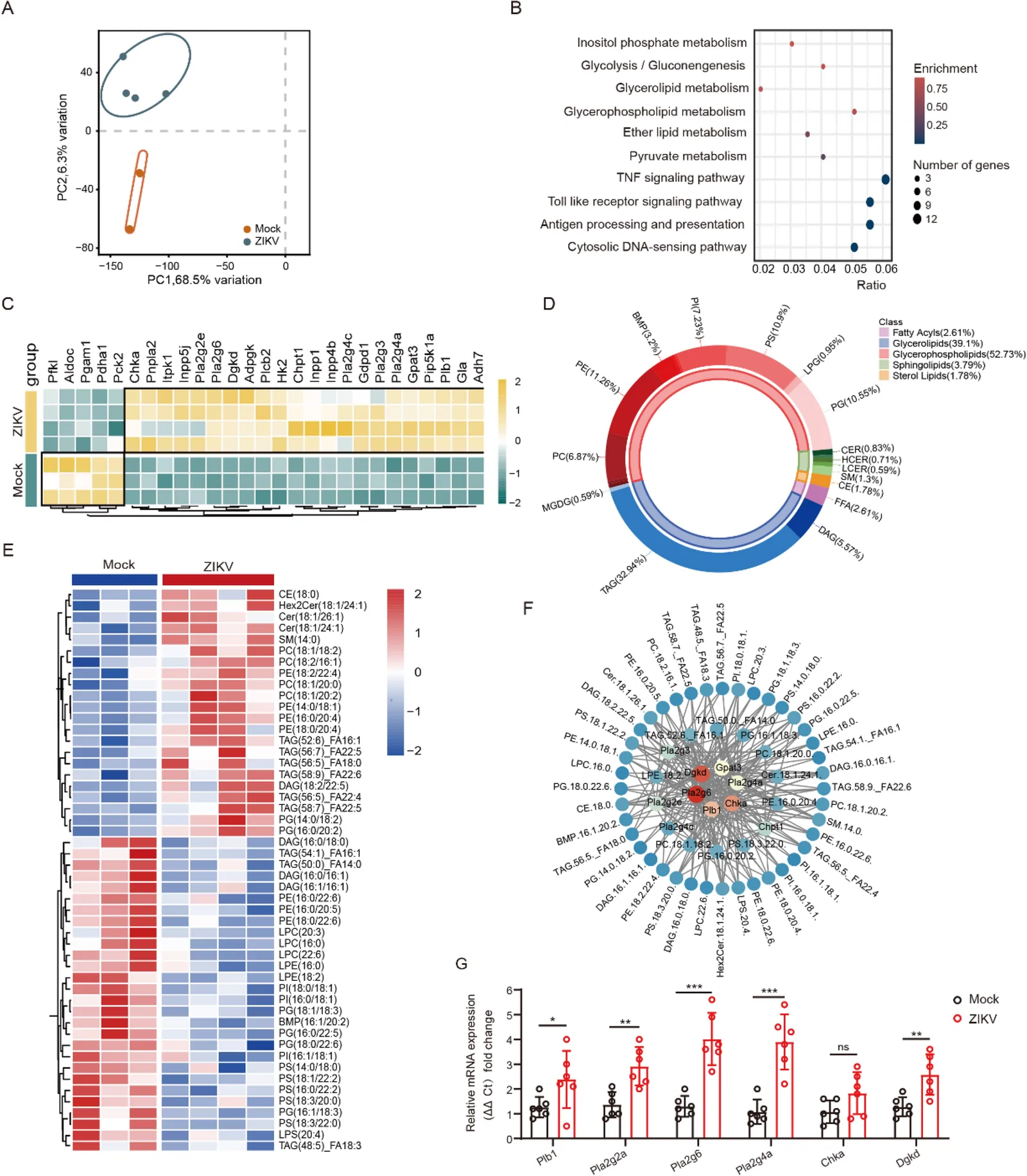
ZIKV infection induces transcriptomic and lipid remodeling associated with iPLA2β activation in neurons. **A** Principal component analysis (PCA) of transcriptomic profiles of control and ZIKV-infected primary mouse cortical neurons at 48 h post-infection. **B** KEGG pathway enrichment analysis of differentially expressed genes (DEGs) after ZIKV infection. **C** Hierarchical clustering of metabolism-related genes in control and ZIKV-infected neurons. **D** Lipid class distribution in primary mouse cortical neurons, as determined by lipidomic analysis. **E** Heatmap of significantly altered lipid species in control and ZIKV-infected neurons. **F** Gene-lipid interaction network within the glycerophospholipid metabolism pathway. **G** Relative mRNA expression of lipid metabolism-related genes in primary mouse cortical neurons following ZIKV infection. *n* = 6. Data are presented as mean ± SEM. Statistical significance was assessed using Mann–Whitney U test. *p < 0.05, **p < 0.01, ***p < 0.001

To investigate the transcriptional and metabolic changes associated with ZIKV infection in neurons, we conducted transcriptomic profiling of primary mouse cortical neurons 48 h post-infection. Differential expression analysis identified 1512 genes that were significantly altered in ZIKV-infected neurons. KEGG pathway enrichment analysis revealed a significant association between these genes and glycerophospholipid metabolism (Fig. 2A–B). Hierarchical clustering analysis underscored the coordinated upregulation of phospholipid remodeling-associated genes, including *Pla2g6* (*iPLA2β*) (Fig. 2C). Untargeted lipidomic profiling revealed that phospholipids were the predominant class of altered lipid species, with significant enrichment of the glycerophospholipid metabolism pathway (Fig. 2D). ZIKV infection resulted in decreased levels of saturated phospholipids, whereas multiple polyunsaturated phospholipid species, including phosphatidylcholine (PC), phosphatidylethanolamine (PE), and phosphatidylglycerol (PG), were increased (Fig. 2E). Despite the observed alterations in phospholipid composition, membrane fluidity in primary neurons was not significantly affected by ZIKV infection (Fig. S1A). To explore the mechanisms underlying phospholipid remodeling and ZIKV infection, a gene–lipid interaction network was generated by integrating differentially expressed genes with lipid species altered in the glycerophospholipid pathway. Within this network, *Pla2g6* emerged as a key node potentially linked to the observed lipid changes (Fig. 2F). qPCR analysis showed that *Pla2g6* mRNA levels were significantly upregulated in ZIKV-infected neurons (Fig. 2G). Consistent with these findings, iPLA2β protein expression was markedly elevated in the cortex of ZIKV-infected neonatal mice (Fig. S1B). Collectively, these data demonstrate coordinated transcriptional and lipid metabolic remodeling in neurons following ZIKV infection, with iPLA2β closely associated with phospholipid alterations.

### ZIKV E protein promotes neuronal iPLA2β upregulation

**Fig. 3:**
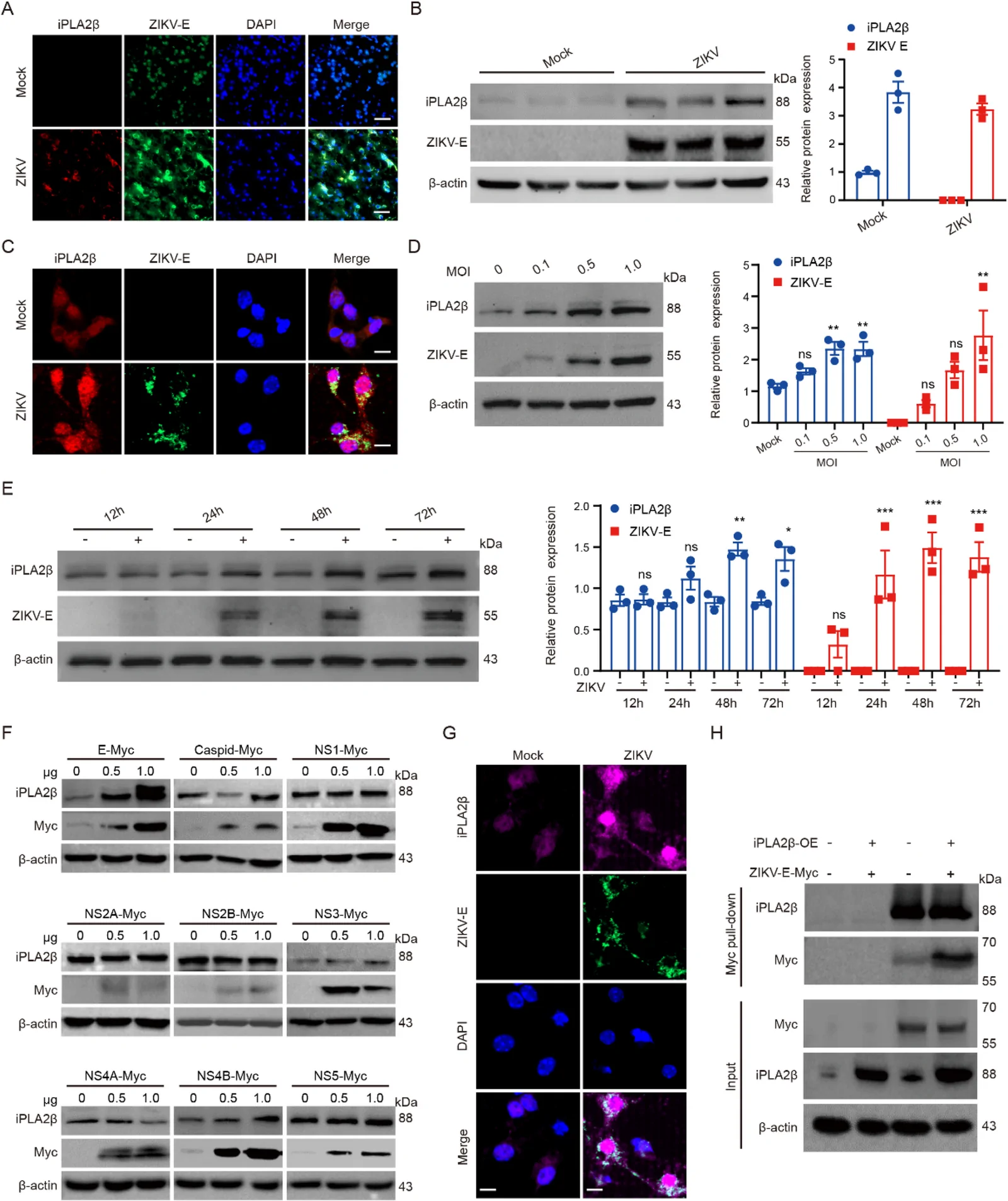
ZIKV E protein promotes neuronal iPLA2β upregulation. **A** Colocalization of iPLA2β and ZIKV E protein in the cortex of neonatal mice infected with ZIKV, as shown in representative immunofluorescence images. Scale bar, 100 μm. **B** Protein expression levels of iPLA2β and ZIKV E protein in the cortex of ZIKV-infected neonatal mice. **C** Colocalization of iPLA2β and ZIKV E protein in primary cortical neurons at 48 h post-infection, as shown by representative immunofluorescence images. Scale bar, 100 μm. **D** Protein expression levels of iPLA2β and ZIKV E protein in primary cortical neurons infected with ZIKV at different multiplicities of infection (MOI). **E** Protein expression levels of iPLA2β and ZIKV E protein in ZIKV-infected primary neurons at different time points post-infection. **F** Protein levels of iPLA2β in SH-SY5Y cells transfected with plasmids expressing individual ZIKV proteins. **G** Expression of iPLA2β in ZIKV-infected SH-SY5Y cells, as shown by representative immunofluorescence images. Scale bar, 100 μm. **H** Interaction between iPLA2β and Myc-tagged ZIKV E protein in SH-SY5Y cells was demonstrated by co-immunoprecipitation and validated by western blotting. Data are presented as mean ± SEM. Statistical significance was determined using the Mann–Whitney U test. *p < 0.05; **p < 0.01; ***p < 0.001

iPLA2β expression was markedly increased following ZIKV infection in multiple systems. In vivo, iPLA2β was significantly upregulated in the cortex of neonatal mice following intracerebroventricular (i.c.v.) ZIKV infection and colocalized with ZIKV E protein (Fig. 3A–B). Similar upregulation and colocalization were observed in primary cortical neurons 48 h post-infection (Fig. 3C). In SH-SY5Y cells, ZIKV infection induced iPLA2β expression in a dose- and time-dependent manner, paralleling the expression pattern of the ZIKV E protein (Fig. 3D–E, Fig. S2A–B). To determine the viral factor responsible for iPLA2β induction, SH-SY5Y cells were transfected with Myc-tagged plasmids expressing individual ZIKV proteins, including structural proteins (C and E) and non-structural proteins (NS1, NS2A, NS2B, NS3, NS4A, NS4B, and NS5). Western blot analysis showed that only the ZIKV E protein, but not the other viral proteins, significantly increased iPLA2β expression (Fig. 3F). Consistently, immunofluorescence analysis of ZIKV-infected SH-SY5Y cells demonstrated that iPLA2β expression was markedly elevated in ZIKV E-positive cells (Fig. 3G). To further explore the interaction between ZIKV E and iPLA2β, structural prediction using AlphaFold suggested a stable interaction. Specifically, domains II and IV of ZIKV E were predicted to interact with multiple domains of iPLA2β, including the N, ankyrin repeat (ANK), and PLP domains, forming an extensive binding interface (Fig. S2C). This interaction was further supported by co-immunoprecipitation (Co-IP) assays, which demonstrated that iPLA2β co-precipitated with Myc-tagged ZIKV E protein in SH-SY5Y cells (Fig. 3H).

### iPLA2β deficiency alleviates ZIKV-induced neuropathology and neuroinflammation

**Fig. 4:**
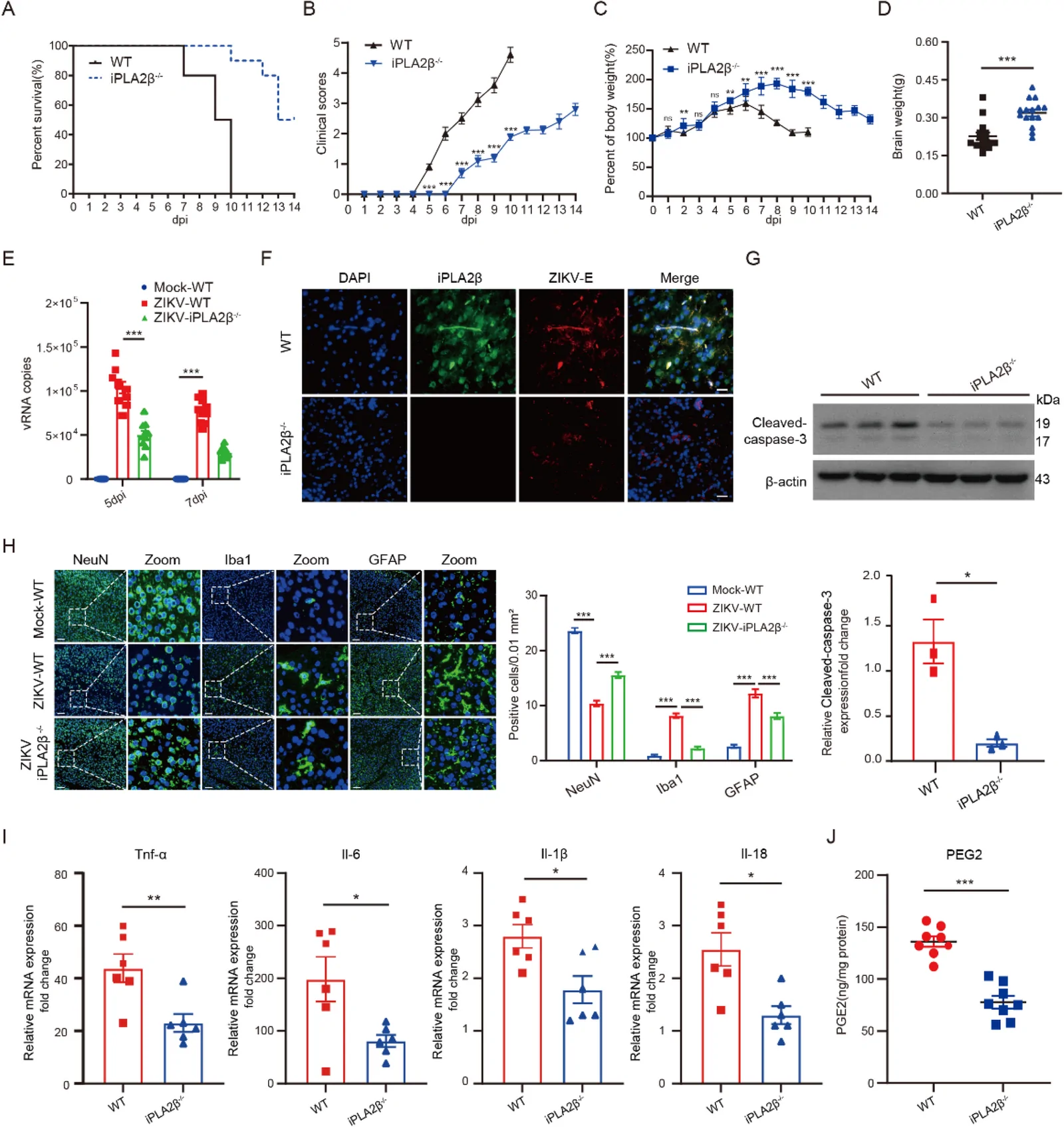
iPLA2β deficiency alleviates ZIKV-induced neuropathology and neuroinflammation. **A** Survival of neonatal iPLA2β^−/−^ and WT mice following intracerebroventricular infection with ZIKV was monitored throughout the experimental period. *n* = 10. **B** Clinical scores of ZIKV-infected iPLA2β^−/−^ and WT mice were evaluated longitudinally during the infection. *n* = 10. **C** Body weight changes in ZIKV-infected iPLA2β^−/−^ and WT mice were measured longitudinally. *n* = 10. **D** Brain weights of ZIKV-infected iPLA2β^−/−^ and WT mice at 7 dpi. *n* = 15. **E** ZIKV RNA levels in the cortex of ZIKV-infected iPLA2β^−/−^ and WT mice at 5 and 7 dpi. *n* = 10. **F** Colocalization of ZIKV E protein and NeuN-positive neurons in the cortex of ZIKV-infected mice at 7 dpi, as shown by representative immunofluorescence images. Scale bar, 100 μm. n = 6. **G** Protein levels of cleaved caspase-3 in the prefrontal cortex of ZIKV-infected iPLA2β^−/−^ and WT mice at 7 dpi. *n* = 3. **H** Neuronal loss and glial activation in the cortex of ZIKV-infected iPLA2β−/− and WT mice at 7 dpi, as shown by representative NeuN, Iba1, and GFAP immunostaining images. Scale bar, 100 μm. n = 6. **I** mRNA expression levels of the pro-inflammatory cytokines *Tnf-α*, *Il-6*, and *Il-1β* in the cortex of ZIKV-infected iPLA2β^−/−^ and WT mice at 7 dpi. *n* = 6. **J** PGE₂ levels in the cortex of ZIKV-infected iPLA2β^−/−^ and WT mice at 7 dpi. *n* = 8. Data are presented as mean ± SEM. Statistical significance was assessed using the Mann–Whitney U test for (B–C, G, I–J) and one-way ANOVA followed by Tukey’s multiple-comparison test for (E, H). *p < 0.05, **p < 0.01, ***p < 0.001

To determine the functional role of iPLA2β in ZIKV infection, neonatal iPLA2β^−/−^ and wild-type (WT) mice were intracerebroventricularly infected with ZIKV on postnatal day 1. Compared with WT controls, ZIKV-infected iPLA2β^−/−^ mice exhibited significantly reduced mortality (Fig. 4A), lower clinical scores (Fig. 4B), and improved body weight gains (Fig. 4C). Brain weight was also significantly increased in iPLA2β^−/−^ mice at 7 dpi (Fig. 4D). Histological analysis further revealed that iPLA2β deficiency attenuated ZIKV-induced cortical pathology, as evidenced by reduced neuronal disorganization, neuronal shrinkage, cytoplasmic rarefaction, and interstitial loosening, compared with ZIKV-infected WT mice (Fig. S3). ZIKV RNA levels at both 5 and 7 dpi were significantly lower in iPLA2β^−/−^ mice than in WT controls (Fig. 4E). Immunofluorescence analysis at 7 dpi revealed extensive colocalization of the ZIKV E protein with NeuN-positive neurons (Fig. 4F). Moreover, neuronal loss and apoptosis in the prefrontal cortex were markedly attenuated in iPLA2β^−/−^ mice (Fig. 4G–H). Reduced neuroinflammatory responses were also observed in iPLA2β-deficient mice, as indicated by decreased Iba1 and GFAP immunoreactivity (Fig. 4H). Consistent with these findings, iPLA2β deficiency significantly reduced the expression of pro-inflammatory cytokines, including *Tnf-α*,* Il-6*, and *Il-1β*, as well as the levels of the inflammatory lipid mediator PGE₂ in the brain following ZIKV infection (Fig. 4I–J).

### iPLA2β deficiency reshapes ZIKV-induced phospholipid remodeling and inflammatory transcriptional programs

**Fig. 5:**
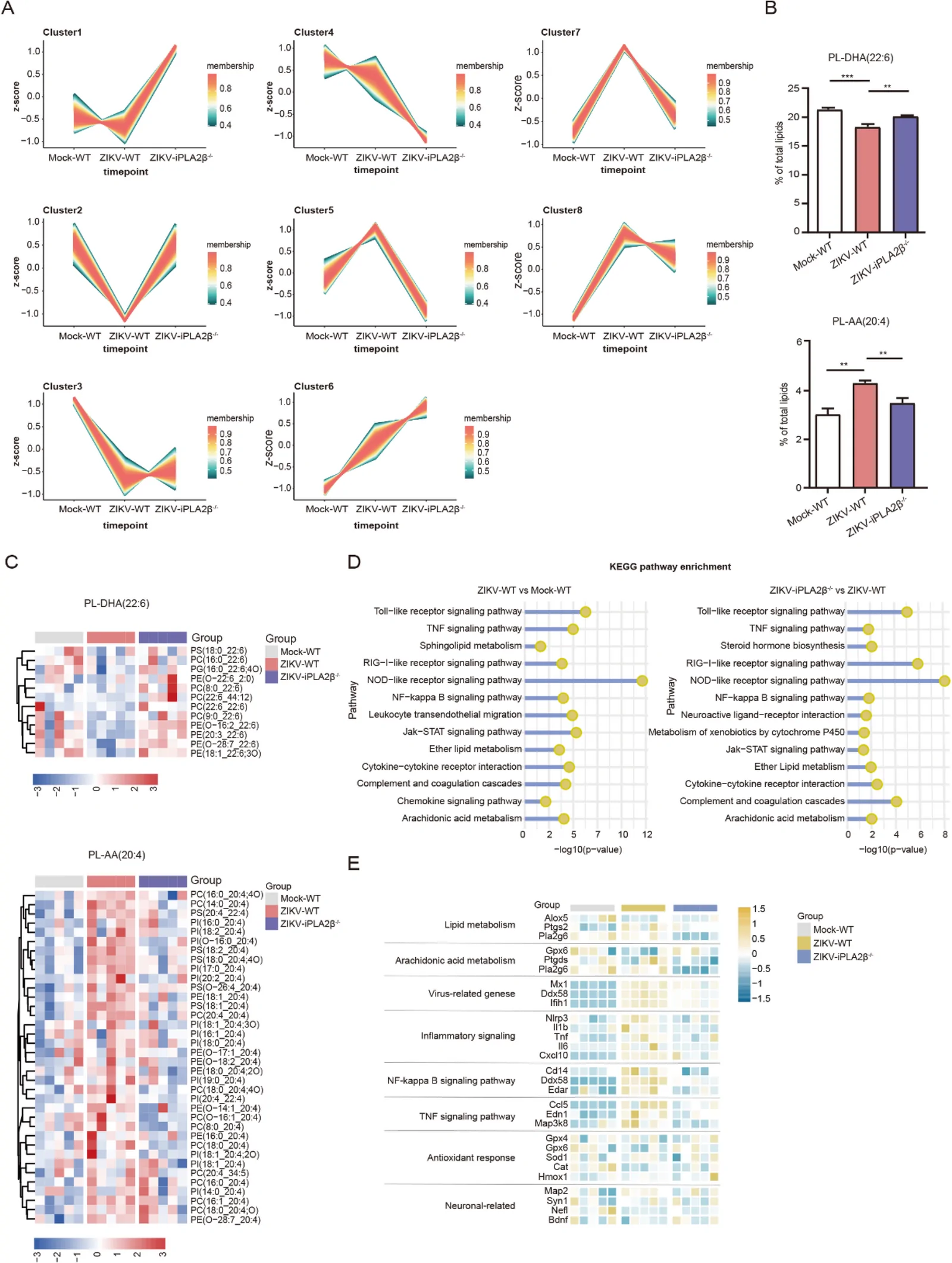
iPLA2β deficiency reshapes ZIKV-induced phospholipid remodeling and inflammatory transcriptional programs. **A** K-means clustering analysis of differentially altered metabolites (DEAMs) across WT-mock, ZIKV-infected WT, and ZIKV-infected iPLA2β^−/−^ mouse cortices. **B** Lipid class distribution of arachidonic acid-containing phospholipids (PL-AA) and docosahexaenoic acid-containing phospholipids (PL-DHA) in WT-mock, ZIKV-infected WT, and ZIKV-infected iPLA2β^−/−^ cortices, as determined by lipidomic analysis. **C** Heatmaps of phospholipid species in WT-mock, ZIKV-infected WT, and ZIKV-infected iPLA2β^−/−^ cortices. **D** KEGG pathway enrichment analysis of differentially expressed genes (DEGs) identified by transcriptomic sequencing in WT-mock, ZIKV-infected WT, and ZIKV-infected iPLA2β^−/−^ cortices. **E** Heatmap of inflammation-related gene expression profiles based on transcriptomic sequencing in WT-mock, ZIKV-infected WT, and ZIKV-infected iPLA2β^−/−^ cortices. Data are presented as mean ± SEM. Statistical analyses were performed using one-way ANOVA followed by post hoc Tukey’s multiple comparisons test where applicable. **p < 0.01, ***p < 0.001

To investigate the effects of iPLA2β deficiency on lipid metabolism and transcriptional responses during ZIKV infection, cortical tissues were collected from WT-mock, ZIKV-infected WT, and ZIKV-infected iPLA2β^−/−^ mice at 7 dpi for integrated lipidomic and transcriptomic analyses. Unsupervised k-means clustering of differentially altered metabolites (DEAMs) identified eight clusters with distinct lipid remodeling patterns across WT-mock, ZIKV-infected WT, and ZIKV-infected iPLA2β^−/−^ cortex (Fig. 5A). Clusters 1 and 2 comprised lipid species that were predominantly decreased following ZIKV infection and increased in iPLA2β^−/−^ mice, and were enriched in docosahexaenoic acid-containing phospholipids (PL-DHA). In contrast, clusters 5, 7, and 8 contained lipid species that were predominantly increased after ZIKV infection and attenuated by iPLA2β deficiency, and were mainly associated with arachidonic acid-containing phospholipids (PL-AA). Consistently, the overall proportion of PL-AA was elevated in ZIKV-infected WT brains but reduced in iPLA2β^−/−^ mice, whereas PL-DHA levels exhibited the opposite trend (Fig. 5B). Heatmap analysis of representative phospholipid species further confirmed increased PL-AA and decreased PL-DHA following ZIKV infection, with these alterations largely reversed in iPLA2β^−/−^ mice (Fig. 5C). Transcriptomic KEGG pathway analysis revealed that iPLA2β deficiency was associated with reduced activation of multiple inflammation- and immune-related pathways, including TNF, NF-κB, Toll-like receptor, NOD-like receptor, and IL-17 signaling pathways, as well as modulation of arachidonic acid metabolism (Fig. 5D). Consistently, genes involved in inflammatory signaling and lipid metabolic processes were broadly upregulated in ZIKV-infected WT mice and attenuated in iPLA2β^−/−^ mice (Fig. 5E). Together, these data indicate that iPLA2β deficiency reshapes ZIKV-induced phospholipid remodeling and inflammatory transcriptional programs, characterized by a shift in arachidonic acid- and docosahexaenoic acid-containing phospholipid species.

### Targeting iPLA2β-mediated lipid dysregulation attenuates ZIKV infection and associated neuroinflammation

**Fig. 6:**
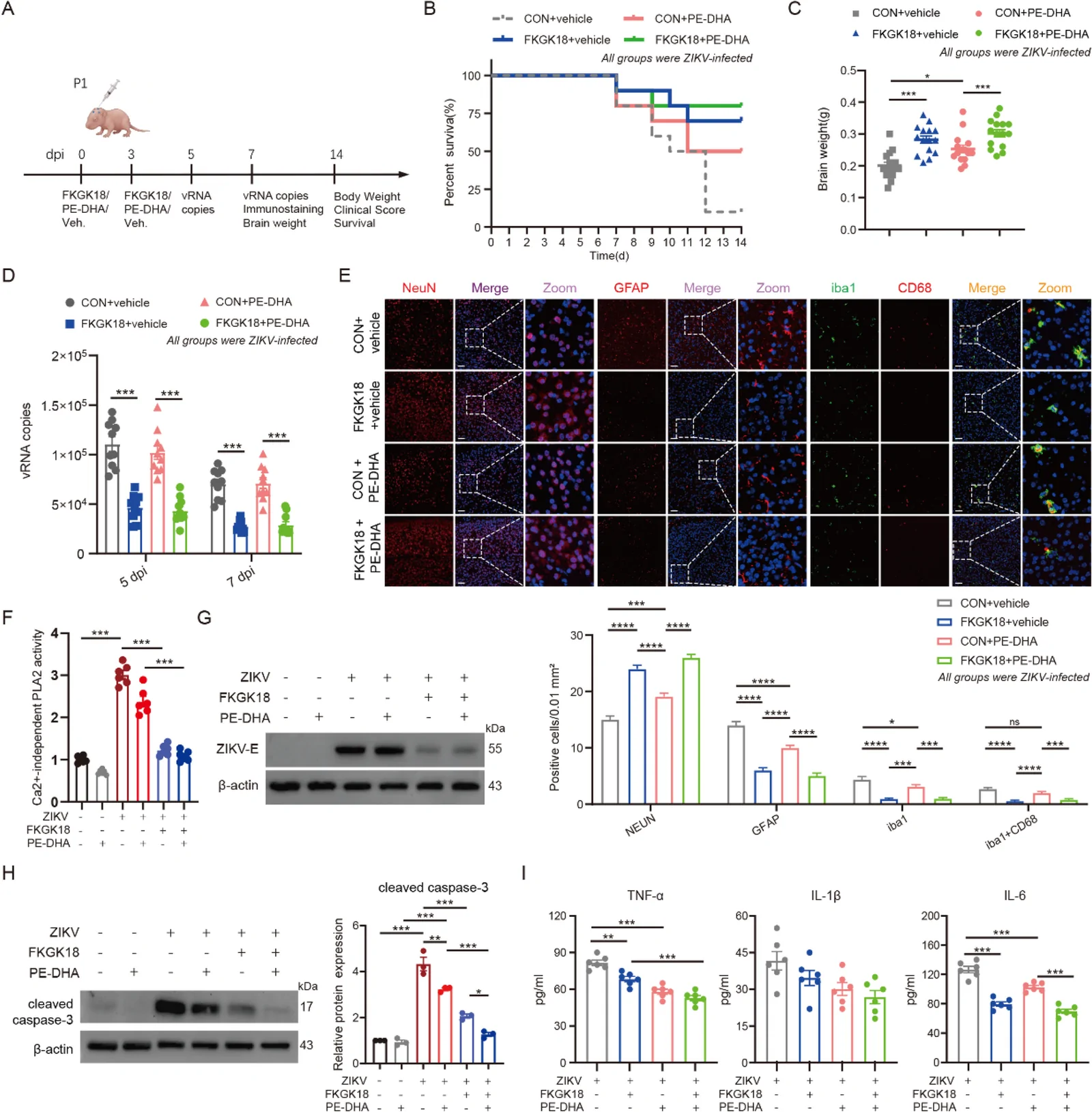
Targeting iPLA2β-mediated lipid dysregulation attenuates ZIKV infection and associated neuroinflammation. **A** Experimental design showing neonatal mice intracerebroventricularly infected with ZIKV and subsequently treated with FKGK18, PE-DHA, or their combination. **B** Survival of ZIKV-infected mice following treatment with FKGK18, PE-DHA, or combined treatment was monitored throughout the experimental period. *n* = 10. **C** Brain weights of ZIKV-infected iPLA2β-/- and WT mice at 7 dpi. *n* = 15. **D** ZIKV RNA levels in the cortex of ZIKV-infected mice at 5 and 7 dpi following treatment with FKGK18, PE-DHA, or their combination. *n* = 10. **E** Neuronal preservation and glial activation in the cortex of ZIKV-infected mice following the indicated treatments at 7 dpi, as shown by representative NeuN, GFAP, and CD68/Iba1 immunofluorescence images. Scale bar, 100 μm. n = 6. **F** Ca²⁺-independent phospholipase A2 activity in primary cortical neurons at 48 h post-infection (hpi). n = 6. **G** ZIKV E protein levels in primary cortical neurons at 48 hpi under the indicated treatment conditions. **H** Protein levels of cleaved caspase-3 in primary cortical neurons at 48 hpi under the indicated treatment conditions. *n* = 3. **I** Protein levels of the pro-inflammatory cytokines TNF-α, IL-6, and IL-1β in primary cortical neurons at 48 hpi, as determined by ELISA. *n* = 6. Data are presented as mean ± SEM. Statistical significance was assessed using one-way ANOVA followed by Tukey’s multiple-comparison test. *p < 0.05, **p < 0.01, ***p < 0.001

To determine the functional relevance of iPLA2β-mediated lipid dysregulation during ZIKV infection, neonatal mice were treated with FKGK18, a selective and reversible inhibitor of iPLA2β [26], PE-DHA, or a combination of these, following intracerebroventricular infection (Fig. 6A). FKGK18 markedly improved survival (Fig. 6B), clinical scores (Fig. S4A), body (Fig. S4B) and brain weights (Fig. 6C), whereas PE-DHA alone exerted modest protective effects. The cortical viral load was also significantly reduced following FKGK18 treatment (Fig. 6D). Immunofluorescence analysis revealed that FKGK18 preserved NeuN-positive neurons and attenuated glial activation, as indicated by reduced GFAP immunoreactivity and decreased CD68 immunoreactivity in Iba1-positive microglia (Fig. 6E). ZIKV infection significantly increased Ca^2+^-independent PLA2 activity in primary cortical neurons, which was attenuated by FKGK18 treatment (Fig. 6F). FKGK18 reduced ZIKV E protein expression in primary cortical neurons (Fig. 6G; Fig. S4C). Both FKGK18 and PE-DHA reduced the levels of cleaved caspase-3, with a more marked effect in the presence of FKGK18 (Fig. 6H; Fig. S4D). No significant differences in membrane fluidity were observed between the experimental groups (Fig. S4E). Consistent with the reduced glial activation, TNF-α and IL-6 levels were significantly decreased following FKGK18 treatment and, to a lesser extent, following PE-DHA supplementation (Fig. 6I). Together, these findings suggest that targeting iPLA2β-mediated lipid dysregulation mitigates ZIKV-induced neuroinflammation and neuronal injury.

### Targeting iPLA2β ameliorates long-term neurological deficits following neonatal ZIKV infection

**Fig. 7:**
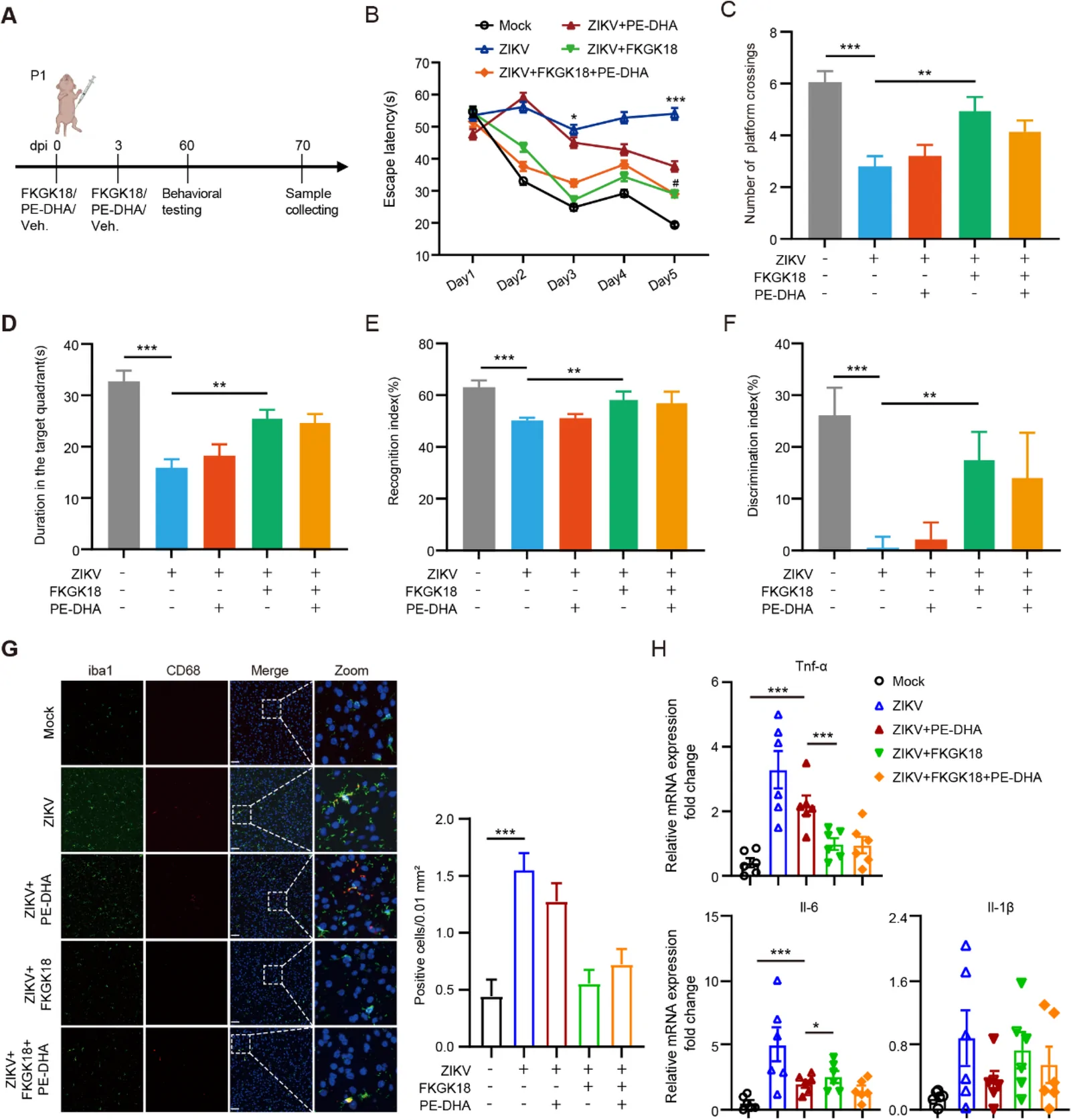
iPLA2β inhibition ameliorates long-term neurological deficits following neonatal ZIKV infection. **A** Schematic representation of the experimental design. Neonatal mice were infected with Zika virus (ZIKV) via intraperitoneal injection and treated with FKGK18, PE-DHA, or their combination, followed by behavioral assessments at 2 months of age. **B** Escape latency in the Morris water maze during the spatial acquisition phase. *n* = 20. **C** Number of platform crossings in the probe trial of the Morris water maze. *n* = 20. **D** Time spent in the target quadrant during the Morris water maze probe test. *n* = 20. **E** Recognition index in the novel object recognition assay. *n* = 20. **F** Discrimination index derived from the novel object recognition test. *n* = 20. **G** Representative immunofluorescence images showing GFAP expression and Iba1/CD68-positive microglia in the cortex. *n* = 6. **H** mRNA expression levels of pro-inflammatory cytokines in the cortex. *n* = 6. Data are presented as mean ± SEM. Statistical significance was assessed using one-way ANOVA followed by post hoc Tukey’s multiple comparisons test. *p < 0.05, **p < 0.01, ***p < 0.001 for FKGK18 + vehicle vs. CON + vehicle; #*p* < 0.01 for FKGK18 + PE-DHA vs. CON + PE-DHA

To evaluate the long-term effects of iPLA2β inhibition on ZIKV-associated neurological impairment, neonatal mice were intraperitoneally infected with ZIKV and treated with FKGK18, PE-DHA, or their combination, followed by behavioral assessment at 2 months of age (Fig. 7A). FKGK18 alone or in combination with PE-DHA partially reduced mortality, improved body and brain weights, and decreased pathological scores in ZIKV-infected mice, whereas PE-DHA alone showed limited protective effects (Fig. S5A–D). Behavioral analyses demonstrated significant cognitive improvement in FKGK18- and FKGK18 + PE-DHA-treated mice. In the Morris water maze, treated animals showed reduced escape latency, more frequent platform crossings, and increased time spent in the target quadrant relative to ZIKV-infected controls (Fig. 7B–D; Fig. S6A). Swimming speed, locomotor activity, and anxiety-related behaviors were comparable among the groups (Fig. S6B–E), excluding nonspecific behavioral effects from the analysis. In the novel object recognition test, FKGK18-treated mice showed higher discrimination and recognition indices than the infected controls (Fig. 7E–F). To further substantiate these findings using a genetic model, we performed long-term behavioral assessments in iPLA2β^−/−^ mice following neonatal ZIKV infection. Consistent with the pharmacological findings, iPLA2β deficiency significantly improved cognitive performance in both the Morris water maze and novel object recognition tests, without affecting swimming speed, locomotor activity, or anxiety-related behaviors (Fig. S7).

Although no significant differences in neuronal numbers in the cortex were observed among groups (Fig. S5E), FKGK18 markedly reduced astrogliosis and microgliosis, as indicated by decreased GFAP immunoreactivity and fewer Iba1/CD68 double-positive microglia (Fig. 7G; Fig. S5F). *Tnf-α* and *Il-6* expression in the prefrontal cortex was significantly reduced following FKGK18 treatment (Fig. 7H). Together, these findings indicate that iPLA2β inhibition alleviates persistent neuroinflammation and improves long-term cognitive outcomes following neonatal ZIKV infection.

## Discussion

ZIKV infection causes severe neurodevelopmental abnormalities and long-term neurological dysfunction, which are closely associated with neuroinflammatory responses that may be triggered by viral replication and host–virus interactions in the developing brain [11, 28]. Although lipid metabolic remodeling is increasingly recognized as a feature of flaviviral infection [23], the mechanisms linking viral replication, phospholipid reprogramming, and persistent neurological injury remain incompletely understood. Here, our findings support a role for iPLA2β as an important host factor associated with ZIKV replication, neuronal phospholipid remodeling, and downstream neuropathological changes. We found that the ZIKV envelope (E) protein interacts with iPLA2β and is associated with increased neuronal iPLA2β expression. This is accompanied by a coordinated shift in phospholipid composition, characterized by increased arachidonic acid–containing species and reduced docosahexaenoic acid–containing phospholipids. Functionally, genetic or pharmacological inhibition of iPLA2β attenuates viral replication, restores phospholipid homeostasis, and is accompanied by reduced neuroinflammatory responses, neuronal injury, and cognitive impairment, suggesting that iPLA2β-dependent phospholipid remodeling may contribute to efficient ZIKV replication and is associated with downstream pathological changes.

It has been previously reported that ZIKV is highly neurotropic and induces robust neuroinflammatory responses in the developing central nervous system [29, 30], such as neural progenitor cell infection with disrupted proliferation and elevated cell death [31, 32]. Consistent with these findings, we confirm that neurons are direct targets of ZIKV infection in vivo and in primary cortical neurons. Neuroinflammation in viral encephalitis has historically been largely attributed to glial activation; however, accumulating evidence indicates that infected neurons also actively contribute to innate immune signaling [33]. In our study, ZIKV-infected neurons exhibited strong activation of the arachidonic acid–prostaglandin E₂ (AA–PGE₂) axis. This inflammatory lipid pathway is an evolutionarily conserved response to viral infection [34, 35]. We found that activation of this pathway was associated with iPLA2β-dependent phospholipid remodeling, which supports a model whereby infected neurons may contribute to the local inflammatory milieu through lipid mediator production.

Transcriptomic and lipidomic analyses identified iPLA2β as a key regulator of ZIKV-associated phospholipid remodeling. As an important enzyme in the Lands cycle, iPLA2β regulates phospholipid turnover, bioactive lipid generation, and inflammatory signaling pathways [36]. Dysregulation of iPLA2β has been associated with neurodegenerative and neuroinflammatory diseases [37], underscoring its importance in neuronal lipid homeostasis. Although basal iPLA2β expression is low in the developing brain at early postnatal stages, ZIKV infection significantly upregulates iPLA2β in neurons, suggesting that viral infection is associated with activation of a lipid remodeling program in developing neurons. This is consistent with the dependence of flaviviruses on host lipid metabolism for the formation of endoplasmic reticulum-derived replication organelles, where membrane composition and curvature critically determine replication efficiency [38]. The marked reduction in viral replication following iPLA2β inhibition suggests that altered phospholipid composition may impair replication organelle formation and/or function, thereby limiting viral amplification [39]. Notably, although phospholipid remodeling was pronounced, no measurable changes in overall membrane fluidity were detected, indicating a selective remodeling of lipid classes rather than global biophysical membrane alterations.

Our results further show that the ZIKV envelope (E) protein interacts with iPLA2β and is associated with increased neuronal iPLA2β expression. Beyond its established roles in viral entry and membrane fusion [40], the E protein may also participate in host metabolic reprogramming, raising the possibility that the E protein contributes to host lipid metabolic remodeling during infection. Our findings suggest that phospholipid remodeling is associated with host–virus interactions during infection rather than representing only a secondary consequence of infection.

Lipidomic analysis revealed that ZIKV-induced iPLA2β-dependent remodeling is characterized by increased arachidonic acid (AA)–containing phospholipids and decreased docosahexaenoic acid (DHA)–containing phospholipids. These alterations reflect a virus-driven metabolic state rather than an intrinsic effect of iPLA2β deficiency. In contrast to steady-state iPLA2β-deficient models, where AA- and DHA-containing phospholipid changes have been reported in juvenile to aged animals due to chronic disruption of phospholipid turnover and compensatory membrane remodeling [41, 42], our study captures a dynamic infection-induced lipid flux state driven by viral replication. These divergent lipid phenotypes reflect fundamentally different metabolic contexts rather than conflicting roles of iPLA2β. Under physiological conditions, iPLA2β maintains basal phospholipid turnover, whereas during ZIKV infection it appears to participate in virus-associated membrane remodeling. Accordingly, iPLA2β appears to facilitate virus-associated lipid remodeling that may favor efficient viral replication rather than determining baseline phospholipid composition, resulting in a PL-AA–dominant and PL-DHA–reduced signature. Consistent with this model, iPLA2β deficiency attenuates infection-driven remodeling and reverses the ZIKV-associated PL-AA/PL-DHA shift in wild-type brains. This restoration of lipid homeostasis is accompanied by reduced production of pro-inflammatory lipid mediators and suppression of NF-κB-, TNF-, and IL-17-associated signaling pathways [43], supporting a model in which iPLA2β-dependent lipid remodeling is associated with efficient viral replication, with inflammatory responses emerging as downstream consequences of altered host–virus metabolic interactions.

Further support for this model comes from pharmacological inhibition studies. In particular, the iPLA2β inhibitor FKGK18 was associated with greater suppression of viral replication, accompanied by reductions in neuroinflammation and neuronal injury, than PE-DHA supplementation, suggesting that targeting upstream metabolic enzymes may exert broader effects [44, 45]. ZIKV replication relies on ER-derived membrane organelles with specific lipid composition and curvature [46], suggesting a strong dependence on host lipid remodeling capacity. This interpretation is consistent with previous findings demonstrating that phospholipase A2 activity is required for efficient replication of the related flavivirus West Nile virus [39]. Given that iPLA2β is localized to the ER membrane system [47], its phospholipid hydrolysis and remodeling activities may contribute to shaping the lipid environment required for viral replication. The reduction in viral burden following iPLA2β inhibition may contribute to reduced activation of inflammatory signaling pathways, thereby providing neuroprotective effects.

Clinically, a subset of ZIKV-infected individuals develops persistent neurological sequelae [48]. We found that iPLA2β inhibition was associated with improved long-term cognitive outcomes. This is consistent with evidence that chronic neuroinflammation is a major determinant of long-term neurological dysfunction following viral encephalitis [49]. The dissociation between neuronal loss and functional recovery suggests that behavioral improvement may result from both reduced viral burden and sustained attenuation of immune–metabolic dysregulation, rather than structural neuronal restoration alone. These findings support further investigation of host lipid metabolic pathways, including iPLA2β, as potential therapeutic targets for ZIKV-associated neurological disease.

This study has several limitations. First, although our data demonstrate an interaction between the ZIKV envelope (E) protein and iPLA2β and show that inhibition of iPLA2β attenuates viral replication and neuropathological changes, the precise molecular mechanisms by which this interaction regulates phospholipid remodeling and viral replication remain to be fully elucidated. Second, our findings are based primarily on neonatal mouse models and primary cortical neurons, which may not fully recapitulate the complexity of human fetal brain development or congenital ZIKV infection. In addition, although FKGK18 showed neuroprotective effects in vivo, the potential off-target effects of pharmacological inhibition and the long-term safety of systemic iPLA2β inhibition were not evaluated in the present study. Finally, while our findings support an association between phospholipid remodeling, viral replication, and neuroinflammatory responses, further mechanistic studies are required to establish the causal relationships among these processes and to determine their respective contributions to neurological outcomes.

## Conclusions

**Fig. 8:**
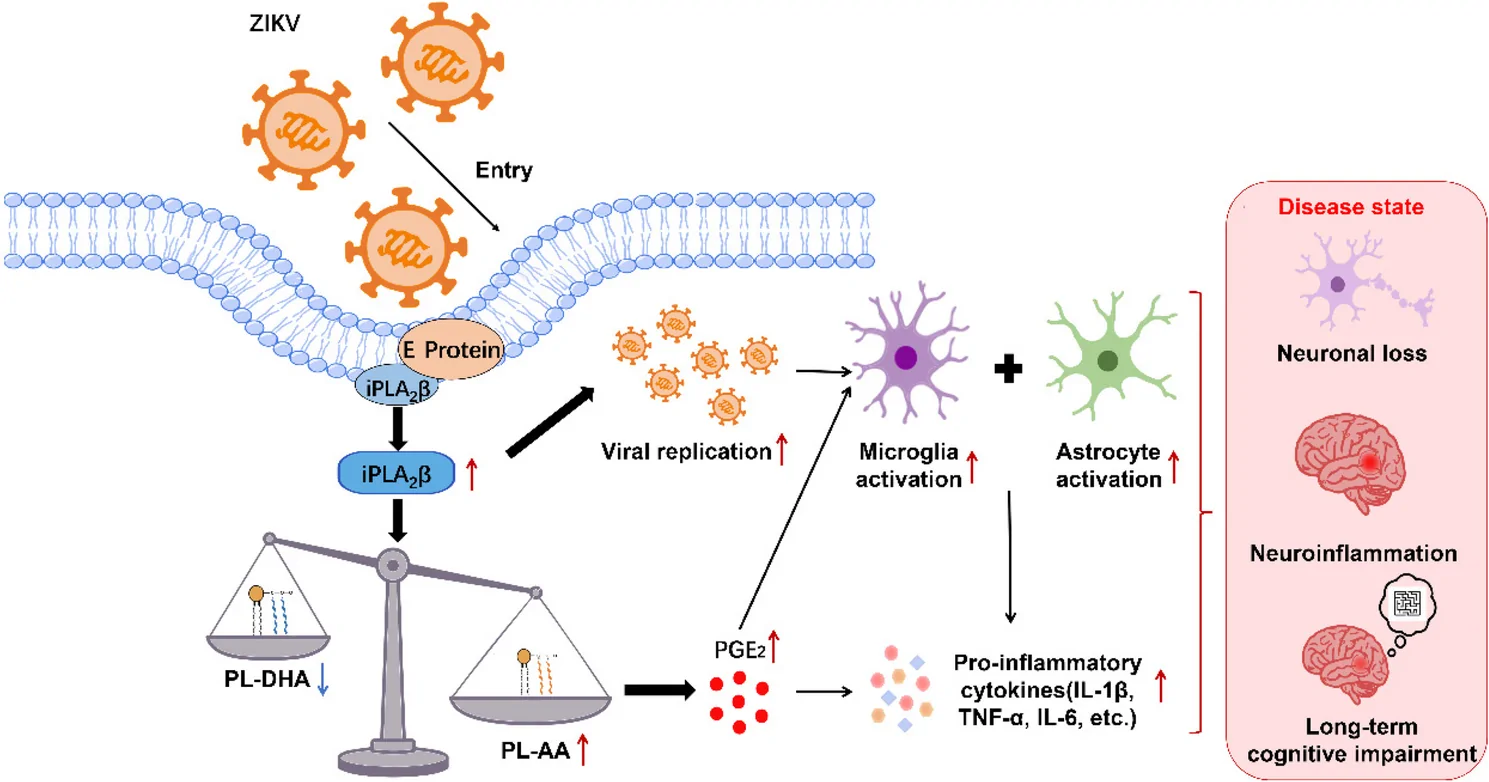
Working model of iPLA2β-dependent phospholipid remodeling in ZIKV-induced neuroinflammation and neurological injury. Zika virus (ZIKV) infection activates neuronal iPLA2β via the viral envelope (E) protein, enhancing viral replication and spread. Upregulation of iPLA2β induces phospholipid remodeling, characterized by decreased PL-DHA and increased PL-AA, leading to elevated PGE₂ production. Increased viral replication and PGE₂ together promote glial activation and pro-inflammatory cytokine expression, resulting in neuronal injury, neuroinflammation, and cognitive impairment

In conclusion, our findings suggest that iPLA2β-dependent phospholipid remodeling is associated with ZIKV replication and neuroinflammatory responses in the developing brain. Our data support an interaction between the ZIKV envelope (E) protein and neuronal iPLA2β, which is accompanied by changes in phospholipid composition during infection. Inhibition of iPLA2β attenuated viral replication and neuropathological changes while restoring phospholipid homeostasis in experimental models (Fig. 8). Together, these findings support a model in which iPLA2β-dependent lipid remodeling is associated with ZIKV-associated neuropathogenesis and suggest that modulation of this pathway warrants further investigation.

## Supplementary Information

Supplementary Material 1.

Supplementary Material 2.

Supplementary Material 3.

Supplementary Material 4.

Supplementary Material 5.

Supplementary Material 6.

Supplementary Material 7.

Supplementary Material 8.

## Data Availability

No datasets were generated or analysed during the current study.
