# Supplementary material for: iPLA2β-dependent phospholipid remodeling drives ZIKV-induced neuroinflammation and neurological injury
Source: J Neuroinflammation. 2026 Jul 20;23:346. doi: 10.1186/s12974-026-03973-2 (PMC13673902; doi:10.1186/s12974-026-03973-2)
Supplement: Supplementary file 1 [file 12974_2026_3973_MOESM1_ESM.pdf]

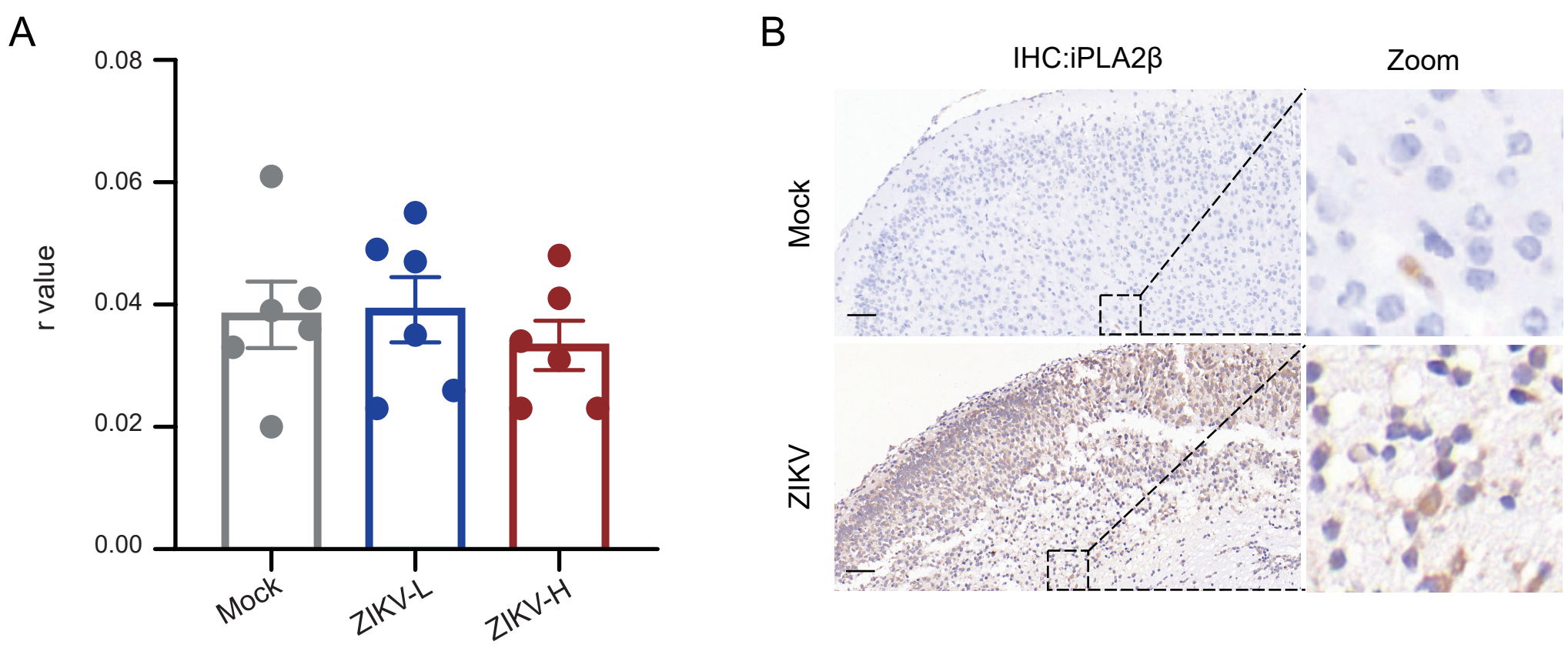

Figure S1. ZIKV infection increases iPLA2β expression but does not significantly alter membrane fluidity in primary neurons

(A) Membrane fluidity of primary neurons following ZIKV infection at low (ZIKV-L) and high (ZIKV-H) multiplicity of infection (MOI). n=6.

(B) Immunohistochemical analysis of iPLA2β expression in neonatal mouse brain following ZIKV infection. Scale bar, 100 μm.
