# Supplementary material for: iPLA2β-dependent phospholipid remodeling drives ZIKV-induced neuroinflammation and neurological injury
Source: J Neuroinflammation. 2026 Jul 20;23:346. doi: 10.1186/s12974-026-03973-2 (PMC13673902; doi:10.1186/s12974-026-03973-2)
Supplement: Supplementary file 2 [file 12974_2026_3973_MOESM2_ESM.pdf]

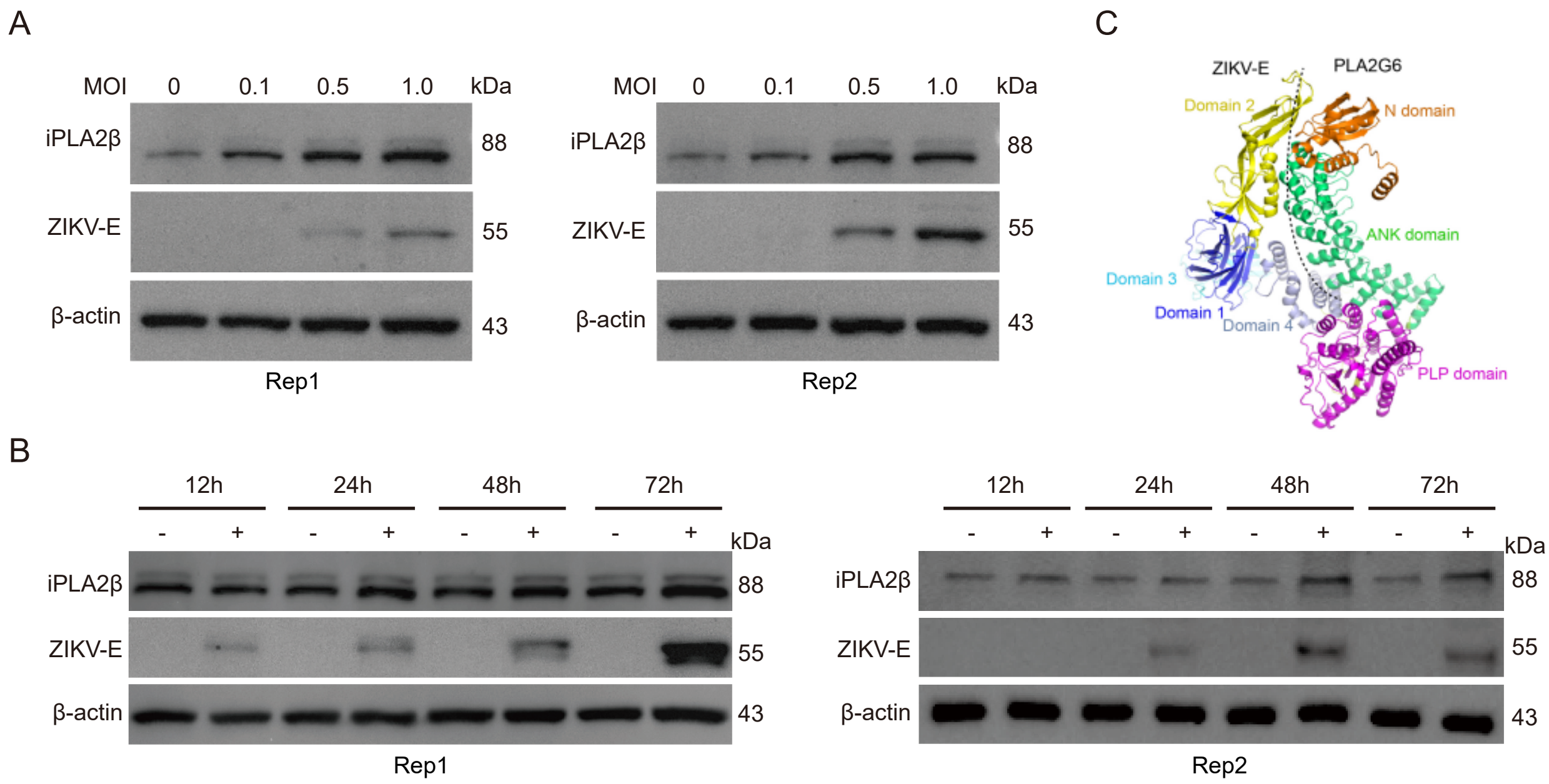

Figure S2. ZIKV E protein induces iPLA2β expression and interacts with iPLA2β

(A) Additional biological replicates of the western blot analysis shown in Fig. 3D.

(B) Additional biological replicates of the western blot analysis shown in Fig. 3E.

(C) Predicted interaction model between ZIKV E protein and iPLA2β generated using AlphaFold. ZIKV E domains II and IV and the corresponding interacting regions within the N domain, ankyrin (ANK) domain, and PLP domain of iPLA2β are indicated.
