# Supplementary material for: iPLA2β-dependent phospholipid remodeling drives ZIKV-induced neuroinflammation and neurological injury
Source: J Neuroinflammation. 2026 Jul 20;23:346. doi: 10.1186/s12974-026-03973-2 (PMC13673902; doi:10.1186/s12974-026-03973-2)
Supplement: Supplementary file 3 [file 12974_2026_3973_MOESM3_ESM.pdf]

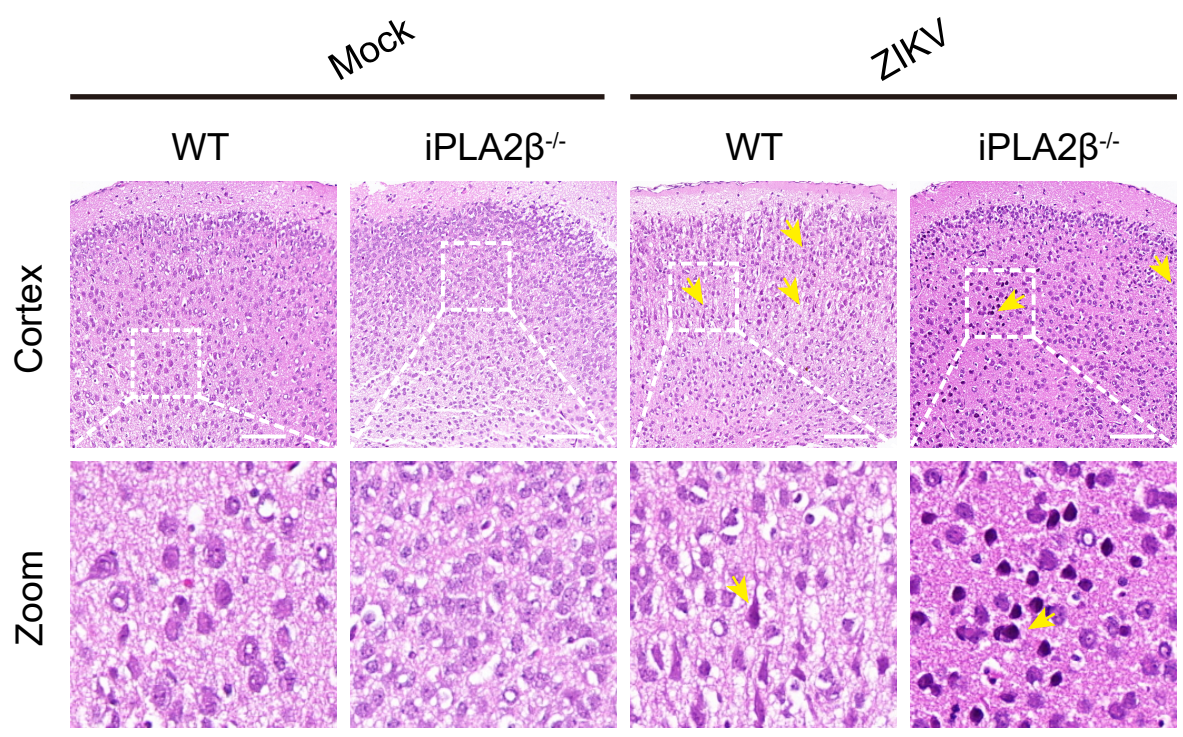


**Figure S3. iPLA2β deficiency attenuates ZIKV-induced brain injury in neonatal mice.** Representative hematoxylin and eosin (H&E) staining of cortical sections from the indicated groups showing neuropathological alterations following ZIKV infection. Scale bar, 100 μm.
