# Supplementary material for: iPLA2β-dependent phospholipid remodeling drives ZIKV-induced neuroinflammation and neurological injury
Source: J Neuroinflammation. 2026 Jul 20;23:346. doi: 10.1186/s12974-026-03973-2 (PMC13673902; doi:10.1186/s12974-026-03973-2)
Supplement: Supplementary file 4 [file 12974_2026_3973_MOESM4_ESM.pdf]

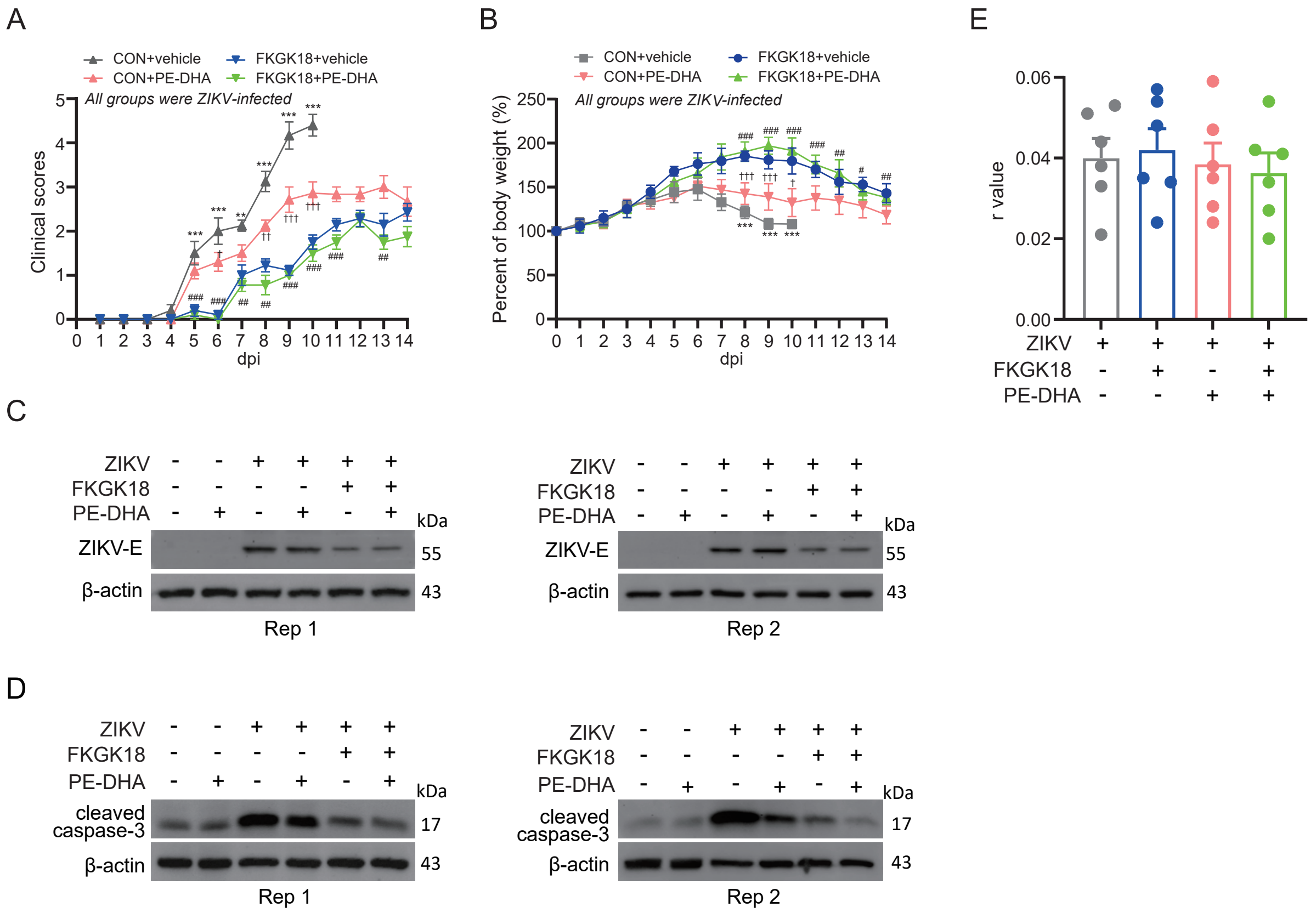

Figure S4. iPLA2β inhibition attenuates ZIKV-induced neuroinflammation and neuronal injury

(A) Clinical scores of neonatal mice following ZIKV infection and treatment with FKGK18, PE-DHA, or their combination. n=10.

(B) Body weight changes in neonatal mice following ZIKV infection and the indicated treatments. n=10.

(C) Additional biological replicates of the western blot analysis shown in Fig. 6F.

(D) Additional biological replicates of the western blot analysis shown in Fig. 6G.

(E) Membrane fluidity analysis of primary cortical neurons. n=6.

Data are presented as mean ± SEM. ***$p < 0.001$ for FKGK18+vehicle vs CON+vehicle; #$p < 0.05$, ##$p < 0.01$, ###$p < 0.001$ for FKGK18+PE-DHA vs CON+PE-DHA; †$p < 0.05$, †††$p < 0.001$ for CON+PE-DHA vs CON vehicle.
