# Supplementary material for: iPLA2β-dependent phospholipid remodeling drives ZIKV-induced neuroinflammation and neurological injury
Source: J Neuroinflammation. 2026 Jul 20;23:346. doi: 10.1186/s12974-026-03973-2 (PMC13673902; doi:10.1186/s12974-026-03973-2)
Supplement: Supplementary file 5 [file 12974_2026_3973_MOESM5_ESM.pdf]

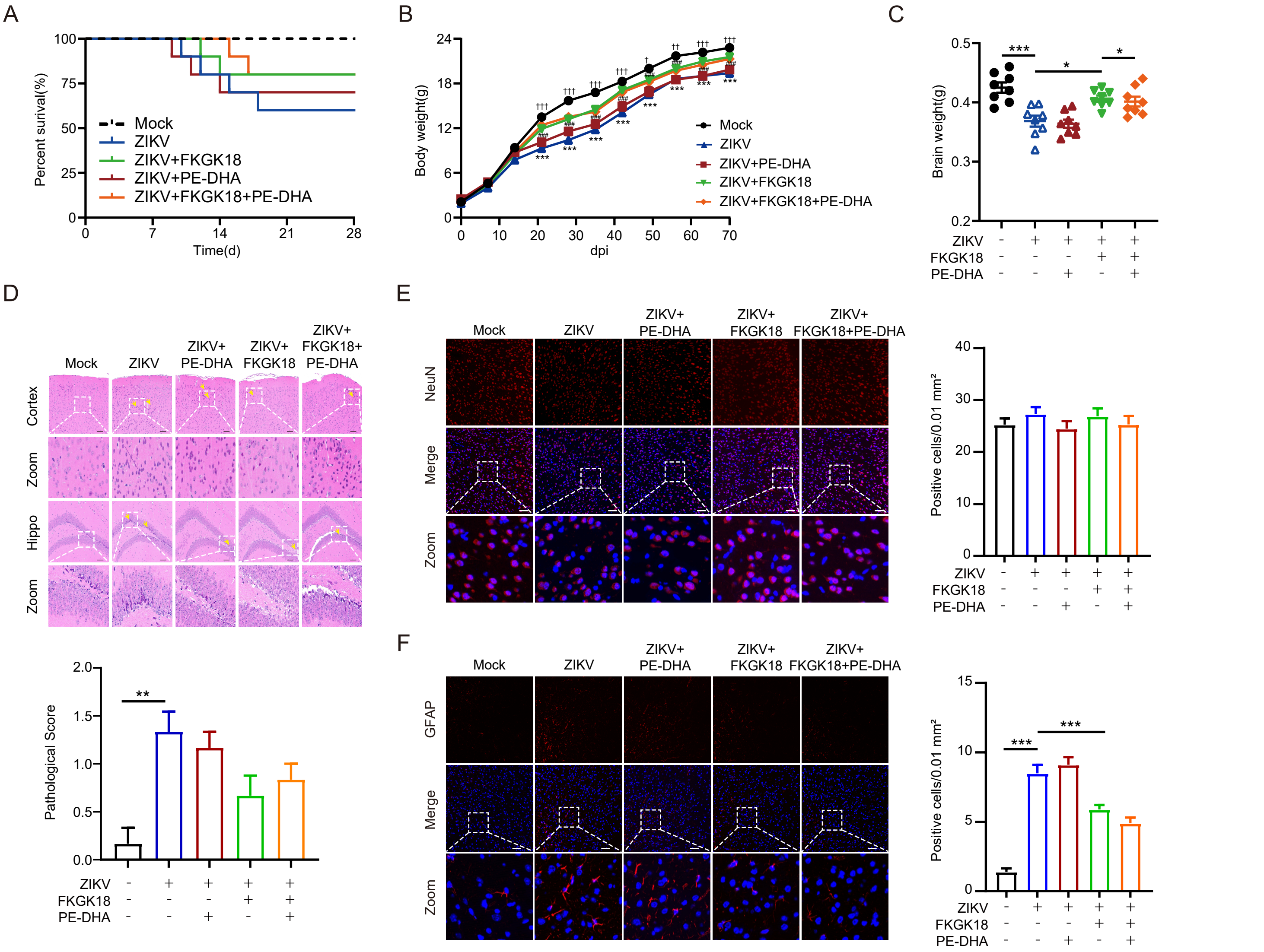

Figure S5. FKGK18 treatment partially alleviates long-term pathological alterations following neonatal ZIKV infection

(A) Survival curves of neonatal mice following intraperitoneal ZIKV infection and treatment with FKGK18, PE-DHA, or their combination. n=10.

(B) Body weight changes of mice during postnatal development. n=20.

(C) Brain weights of mice at 2 months of age. n=8.

(D) Representative H&E staining images of the cortex and hippocampus, and pathological scoring of mice in different experimental groups. n=3.

(E) Representative immunofluorescence images of NeuN staining in the prefrontal cortex of adult mice following neonatal ZIKV infection and treatment. Scale bar, 100 μm. n=6.

(F) Representative immunofluorescence images of GFAP staining in the prefrontal cortex of adult mice following neonatal ZIKV infection and treatment. Scale bar, 100 μm. n=6.

Data are presented as mean ± SEM. ****p* < 0.001 for FKGK18 + vehicle vs CON + vehicle; ### *p* < 0.001 for FKGK18 +PEDHA vs CON + PE-DHA; † *p* < 0.05, †† *p* < 0.01, ††† *p* < 0.001 for CON + PE-DHA vs CON + vehicle.
