# Supplementary material for: iPLA2β-dependent phospholipid remodeling drives ZIKV-induced neuroinflammation and neurological injury
Source: J Neuroinflammation. 2026 Jul 20;23:346. doi: 10.1186/s12974-026-03973-2 (PMC13673902; doi:10.1186/s12974-026-03973-2)
Supplement: Supplementary file 6 [file 12974_2026_3973_MOESM6_ESM.pdf]

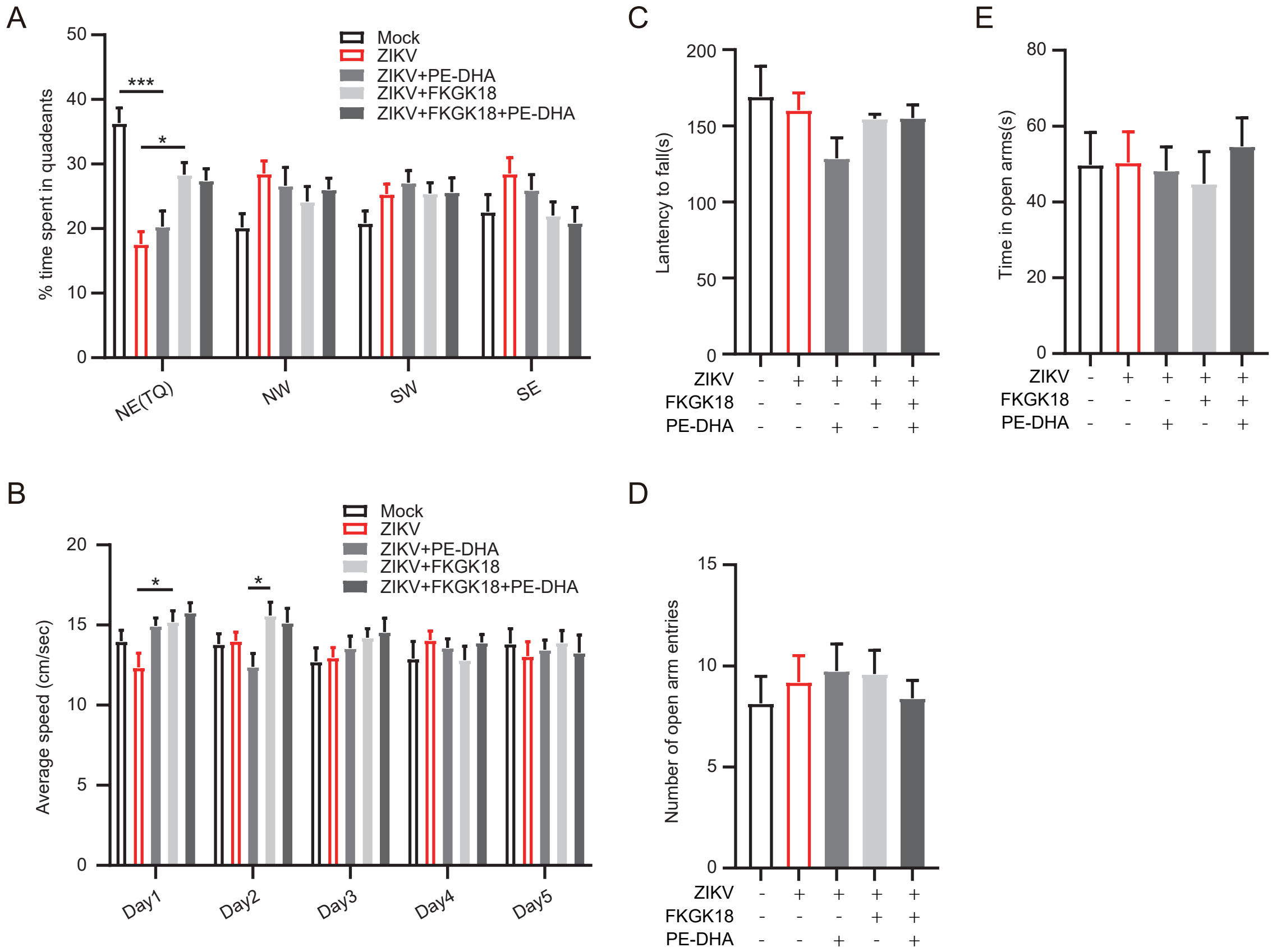

Figure S6. Behavioral control analyses in mice following neonatal ZIKV infection

(A) The percentage of time mice spent in each quadrant during the probe trial of the Morris water maze. n=20.

(B) The average speed of mice during the acquisition phase of the Morris water maze. n=20.

(C) The latency time of mice to fall from the rotating rod during the rotarod test. n=20.

(D) The number of times mice entries the open arm in elevated plus maze. n=20.

(E) The time spent by mice in the open arms in elevated plus maze. n=20.

NE (TQ), northeast quadrant (target quadrant); NW, northwest quadrant; SW, southwest quadrant; SE, southeast quadrant. Data are presented as mean±SEM. Statistical significance was determined using the Kruskal–Wallis test followed by Dunn's multiple-comparisons test.* p < 0.05, *** p < 0.001.
