# Supplementary material for: iPLA2β-dependent phospholipid remodeling drives ZIKV-induced neuroinflammation and neurological injury
Source: J Neuroinflammation. 2026 Jul 20;23:346. doi: 10.1186/s12974-026-03973-2 (PMC13673902; doi:10.1186/s12974-026-03973-2)
Supplement: Supplementary file 7 [file 12974_2026_3973_MOESM7_ESM.pdf]

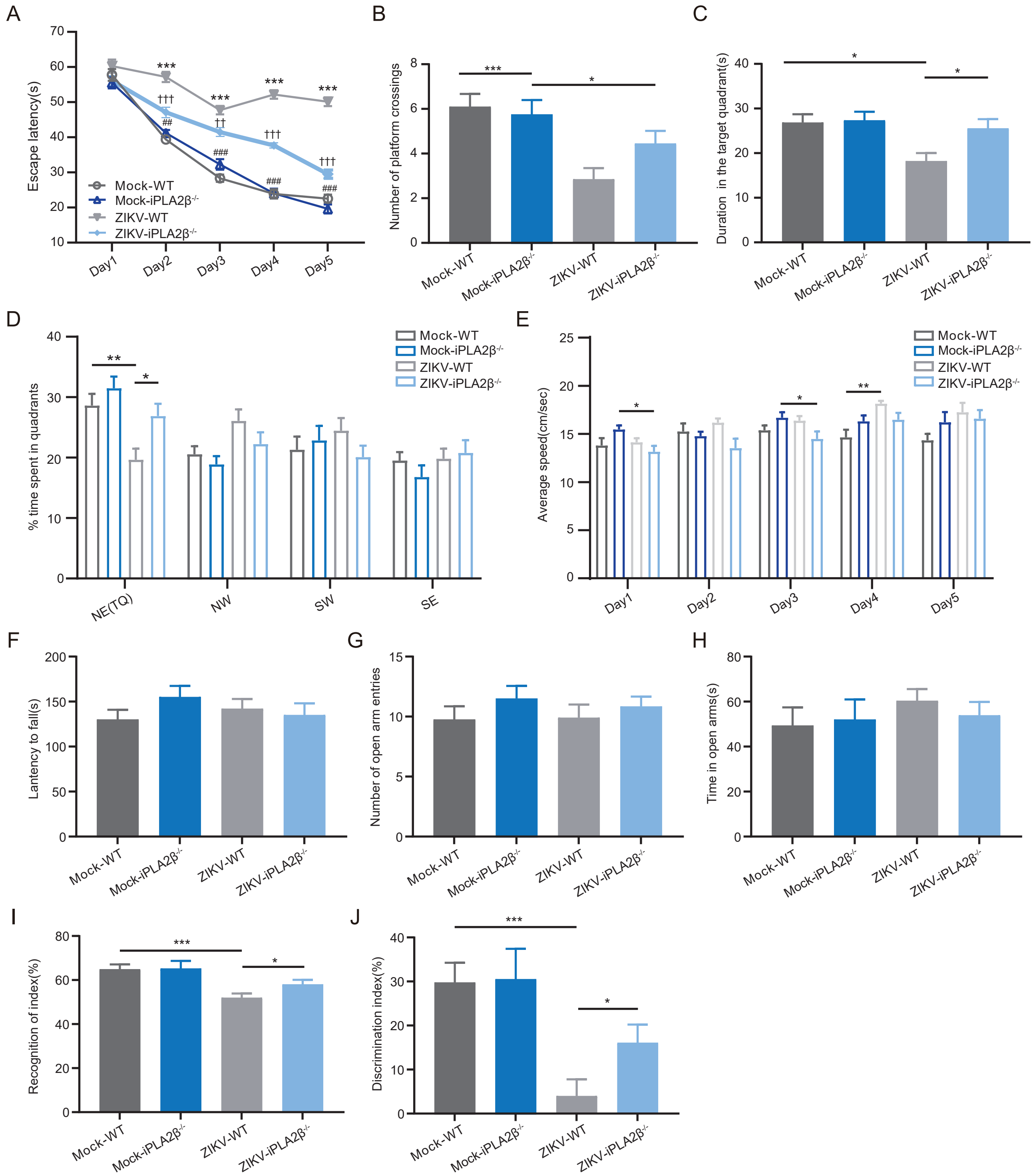

Figure S7. Long-term behavioral assessment of iPLA2β$^{-/-}$ mice following neonatal ZIKV infection

(A) Escape latency in the Morris water maze during the spatial acquisition phase. n=20.
(B) Number of platform crossings in the probe trial of the Morris water maze. n=20.
(C) Time spent in the target quadrant during the Morris water maze probe test. n=20.
(D) The percentage of time mice spent in each quadrant during the probe trial of the Morris water maze. n=20.
(E) The average speed of mice during the acquisition phase of the Morris water maze. n=20.
(F) The latency time of mice to fall from the rotating rod during the rotarod test. n=20.
(G) The number of times mice entries the open arm in elevated plus maze. n=20.
(H) The time spent by mice in the open arms in elevated plus maze. n=20.
(I) Recognition index in the novel object recognition assay. n=20.
(J) Discrimination index derived from the novel object recognition test. n=20.

NE (TQ), northeast quadrant (target quadrant); NW, northwest quadrant; SW, southwest quadrant; SE, southeast quadrant. Data are presented as mean ± SEM. ***p < 0.001 for ZIKV-WT vs Mock-WT; ##p <0.01, ###p <0.001 for ZIKV-iPLA2β$^{-/-}$ vs Mock-iPLA2β$^{-/-}$; ††p < 0.01, †††p < 0.001 for ZIKV-iPLA2β$^{-/-}$ vs ZIKV-WT.
