# Supplementary material for: iPLA2β-dependent phospholipid remodeling drives ZIKV-induced neuroinflammation and neurological injury
Source: J Neuroinflammation. 2026 Jul 20;23:346. doi: 10.1186/s12974-026-03973-2 (PMC13673902; doi:10.1186/s12974-026-03973-2)
Supplement: Supplementary file 8 [file 12974_2026_3973_MOESM8_ESM.pdf]

Fig 1

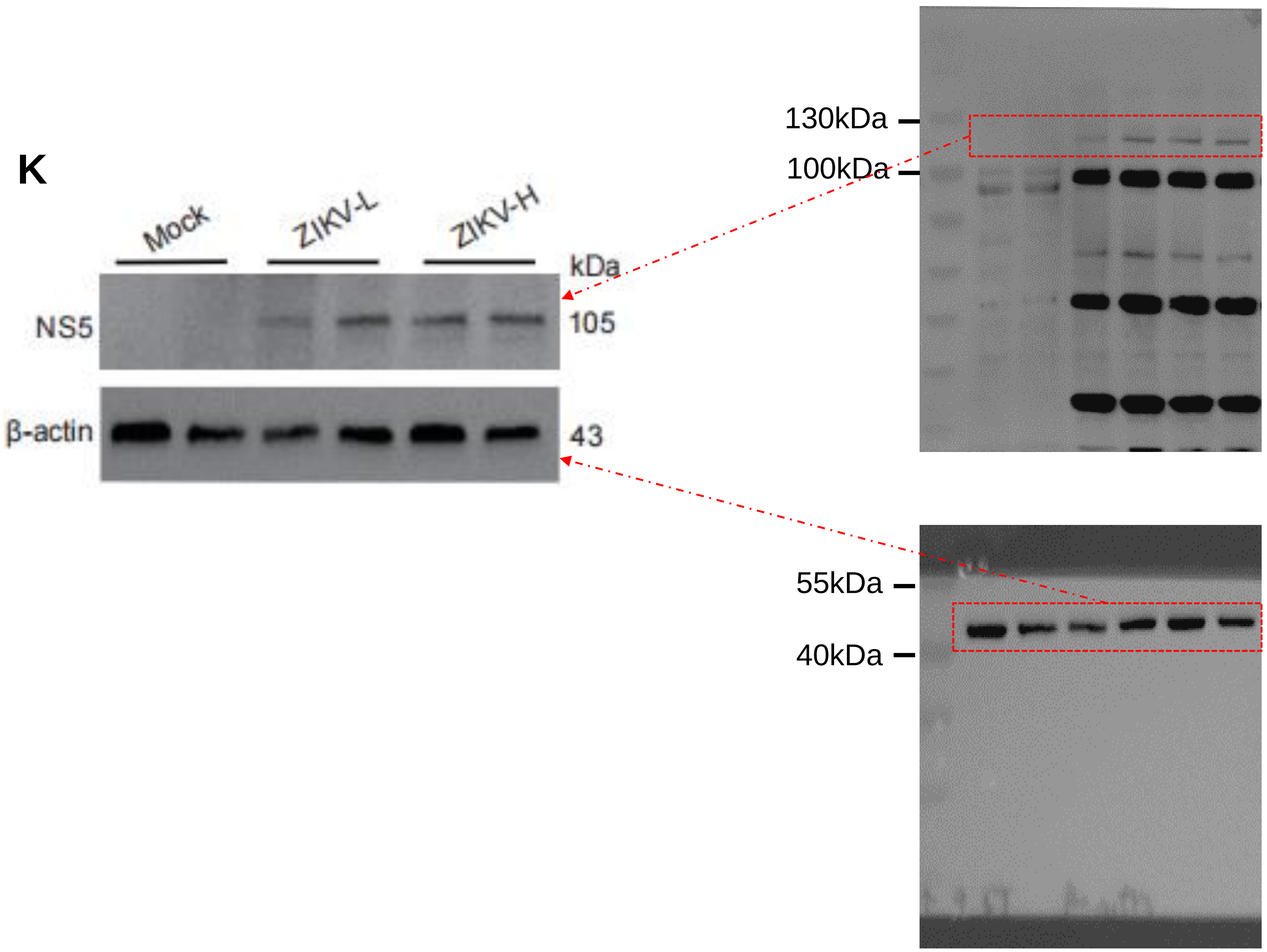
K
Mock
ZIKV-L
ZIKV-H
kDa
NS5
105
β-actin
43
130kDa
100kDa
55kDa
40kDa

Fig 3

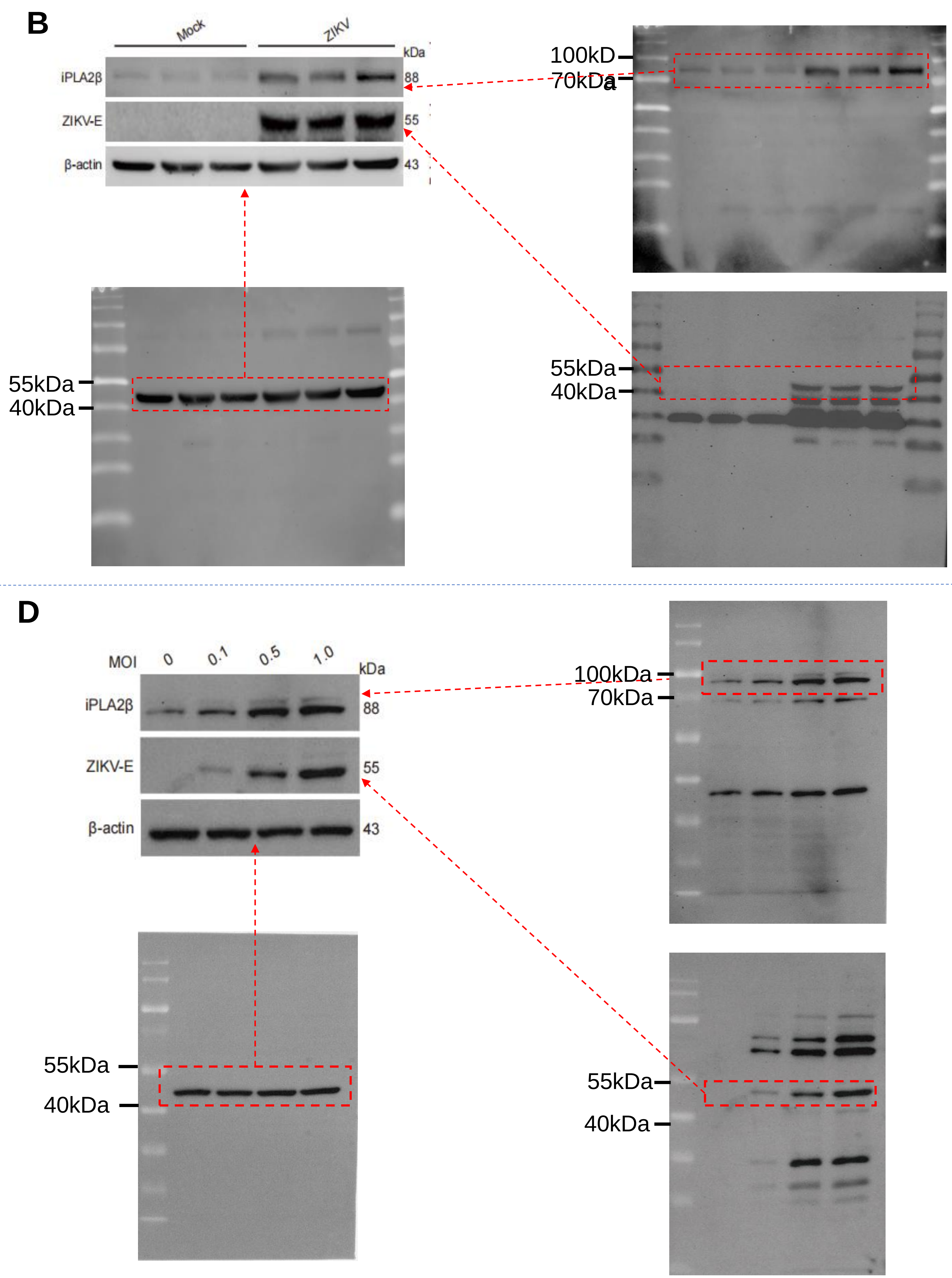

B
Mock
ZIKV
kDa
iPLA2β
88
ZIKV-E
55
β-actin
43
100kD
70kDa
55kDa
40kDa
55kDa
40kDa
D
MOI
0
0.1
0.5
1.0
kDa
iPLA2β
88
ZIKV-E
55
β-actin
43
100kDa
70kDa
55kDa
40kDa
55kDa
40kDa

Fig 3

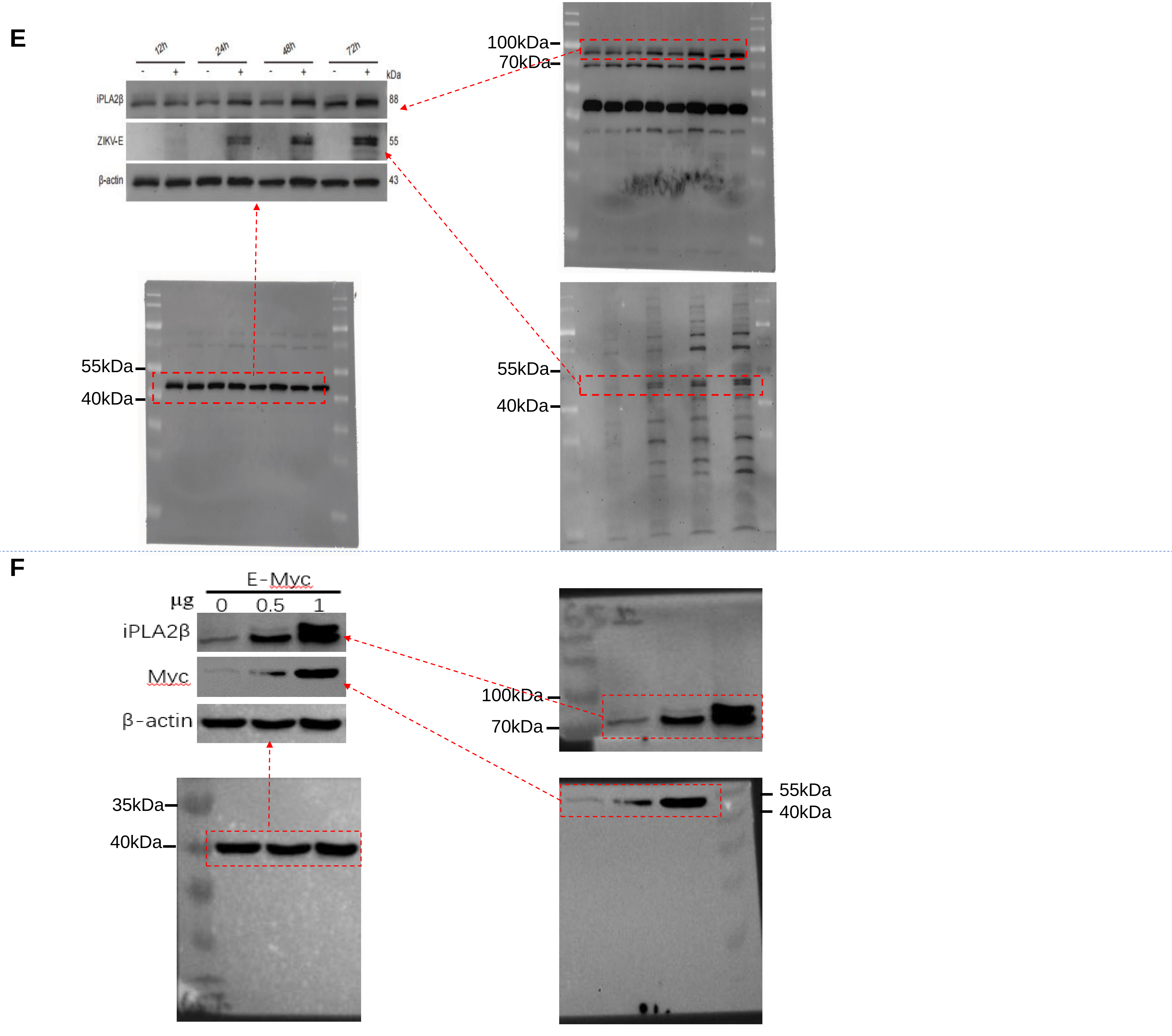

E
12h
24h
48h
72h
kDa
iPLA2β
ZIKV-E
β-actin
88
55
43
100kDa
70kDa
55kDa
40kDa
F
E-Myc
μg
0
0.5
1
iPLA2β
Myc
β-actin
35kDa
40kDa
100kDa
70kDa
55kDa

Fig 3

F

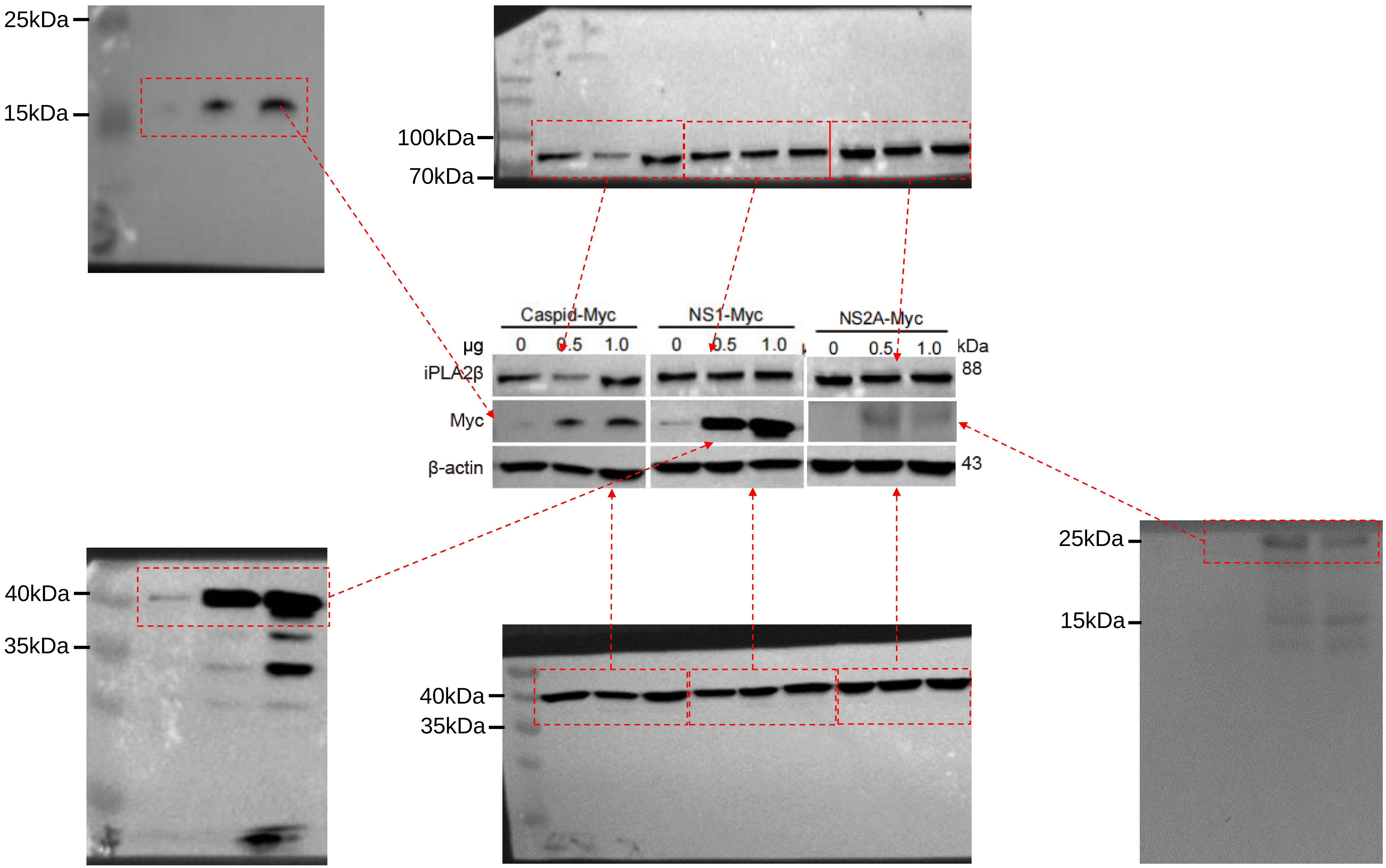

25kDa
15kDa
100kDa
70kDa
Caspid-Myc
NS1-Myc
NS2A-Myc
μg
0
0.5
1.0
0
0.5
1.0
0
0.5
1.0
kDa
iPLA2β
88
Myc
β-actin
43
40kDa
35kDa
40kDa
35kDa
25kDa
15kDa

Fig 3

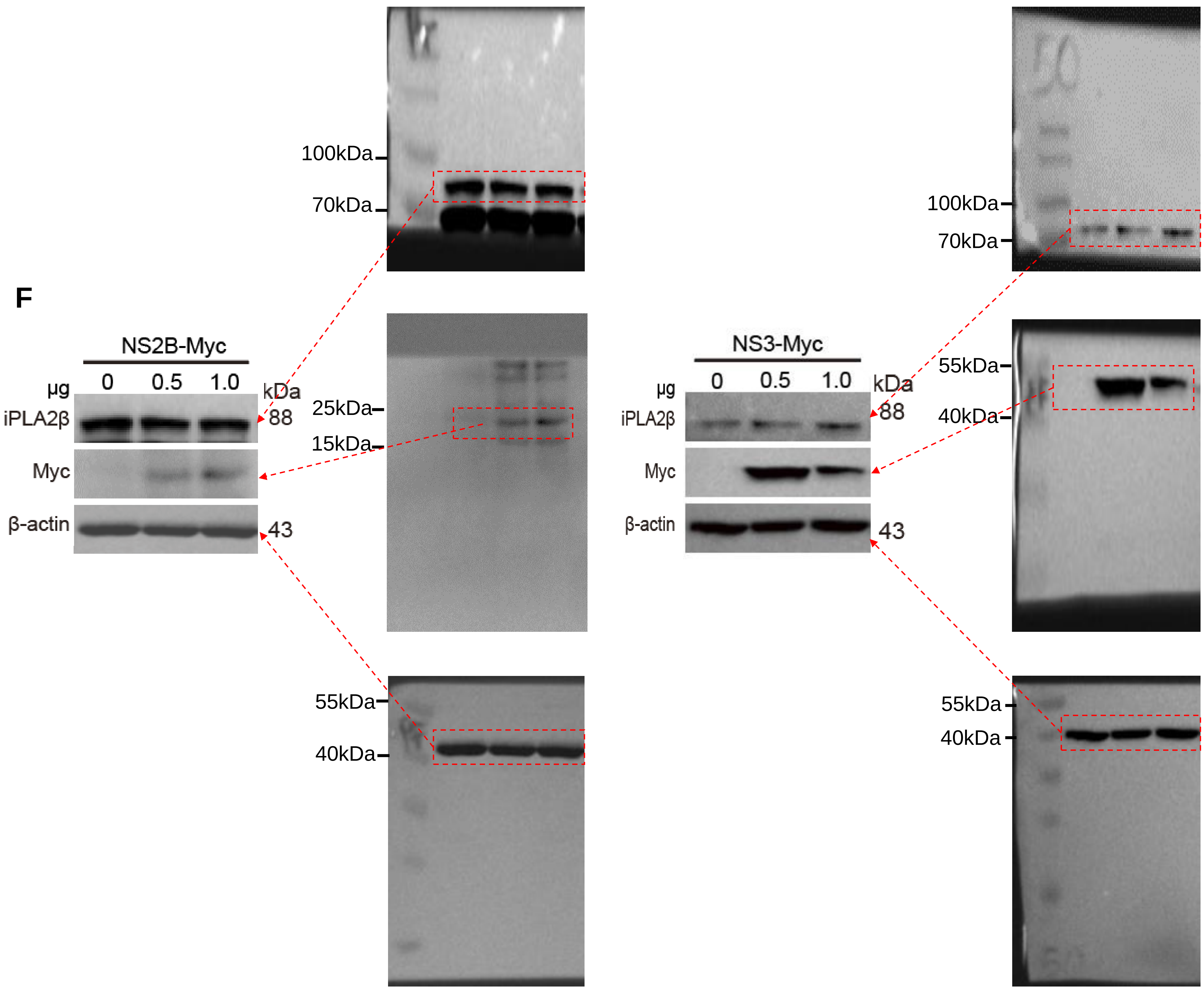

F
NS2B-Myc
μg
0
0.5
1.0
kDa
iPLA2β
88
Myc
β-actin
43
100kDa
70kDa
25kDa
15kDa
55kDa
40kDa
NS3-Myc
μg
0
0.5
1.0
kDa
iPLA2β
88
Myc
β-actin
43
100kDa
70kDa
55kDa
40kDa
55kDa
40kDa

Fig 3

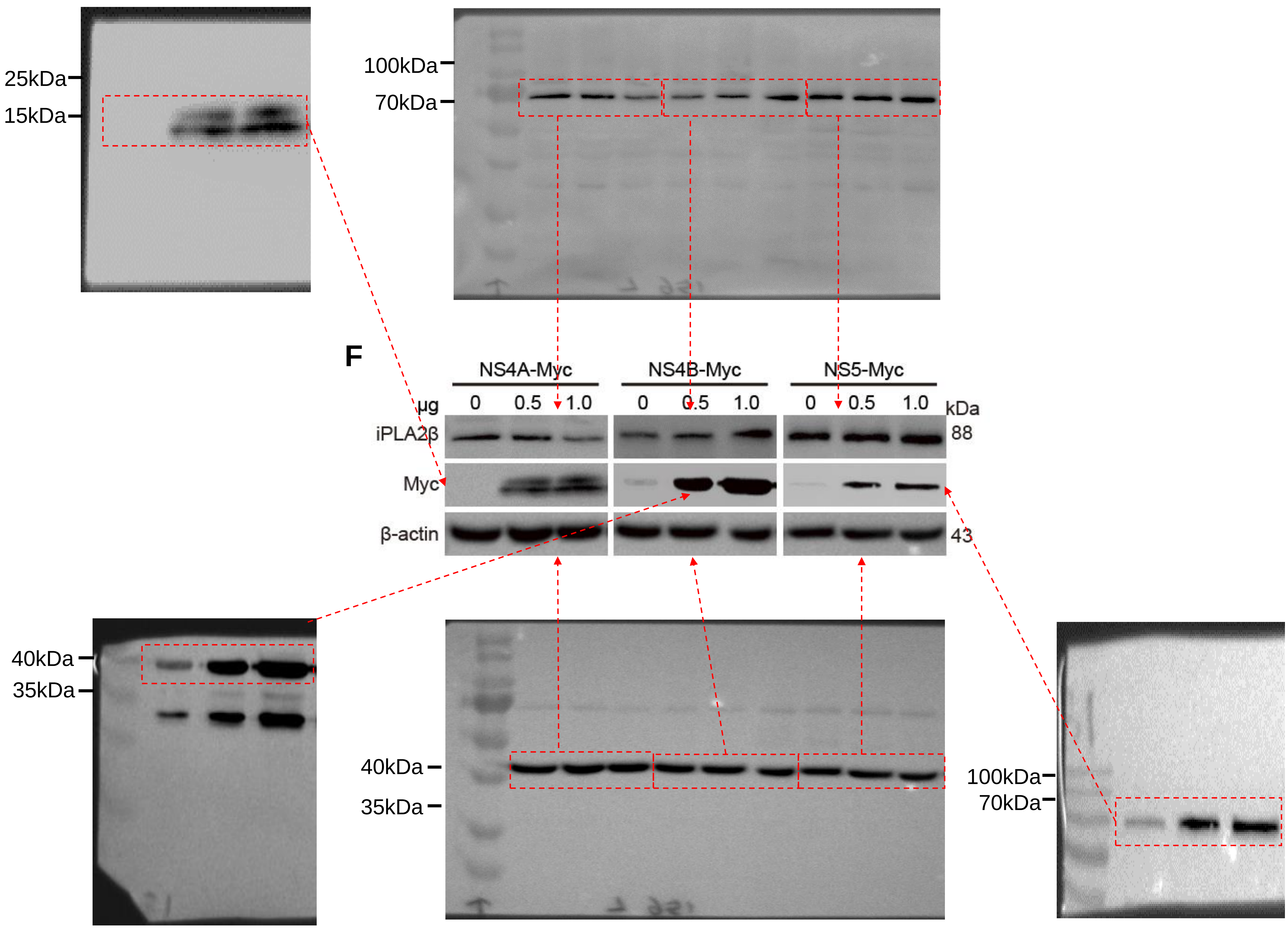

25kDa
15kDa
100kDa
70kDa
F
NS4A-Myc
NS4B-Myc
NS5-Myc
μg
0
0.5
1.0
kDa
iPLA2β
88
Myc
β-actin
43
40kDa
35kDa
40kDa
35kDa
100kDa
70kDa

Fig 3

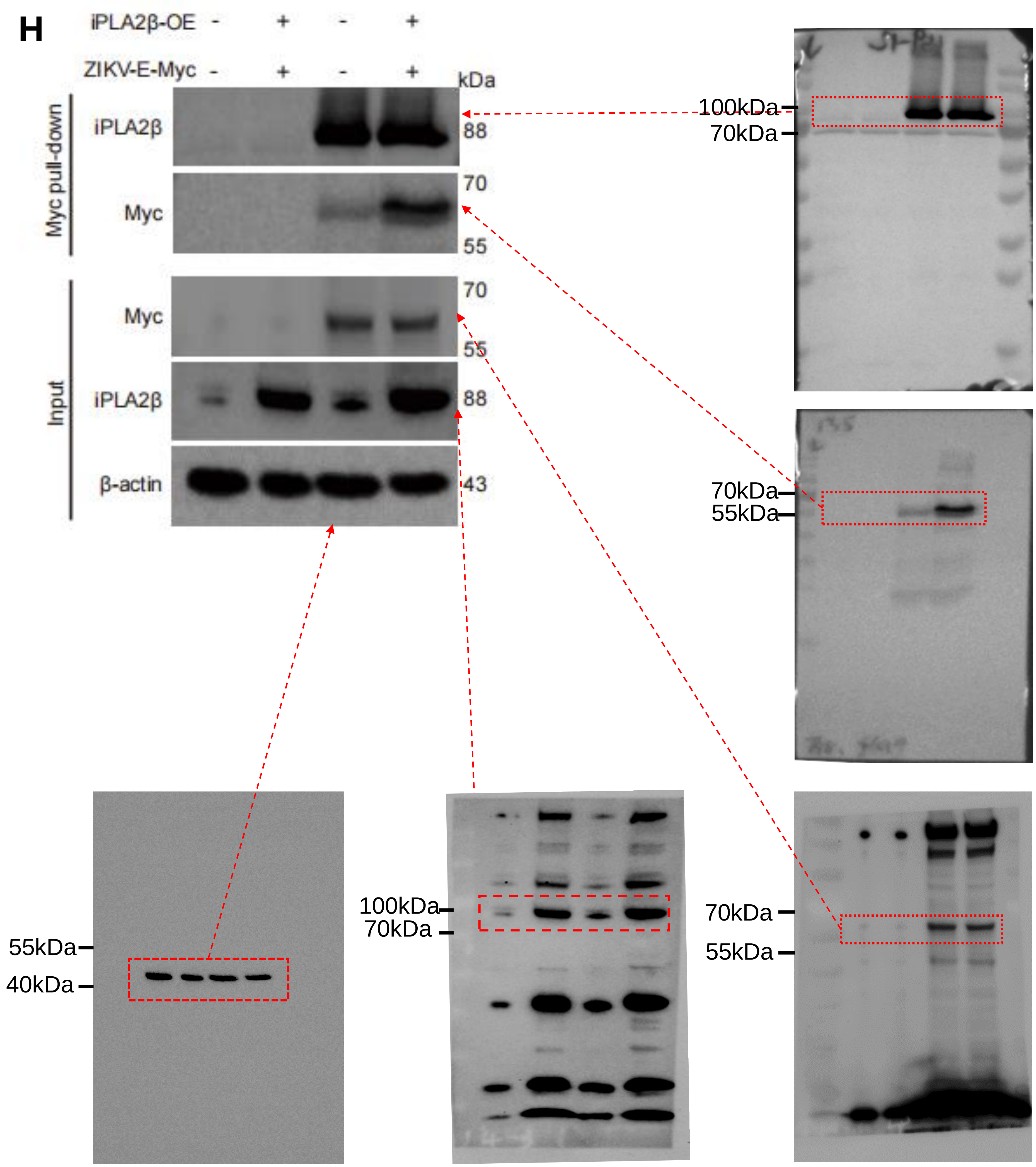

H
iPLA2β-OE - + - +
ZIKV-E-Myc - + - +
kDa
Myc pull-down
iPLA2β
88
Myc
70
55
Input
Myc
70
55
iPLA2β
88
β-actin
43
100kDa
70kDa
70kDa
55kDa
55kDa
40kDa
100kDa
70kDa
70kDa
55kDa

Fig 4

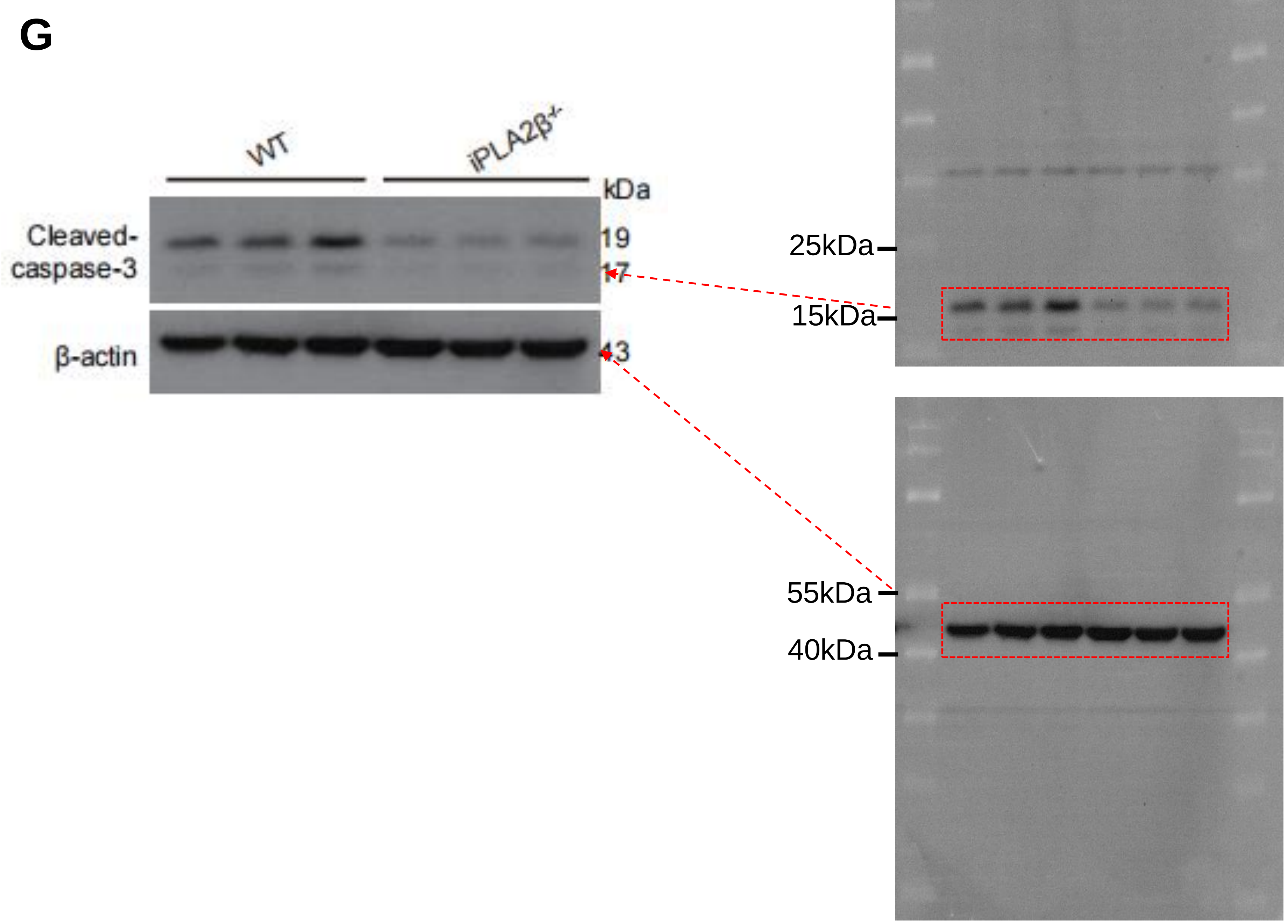
G
WT
iPLA2β-/-
kDa
Cleaved-caspase-3
19
17
β-actin
43
25kDa
15kDa
55kDa
40kDa

Fig 6

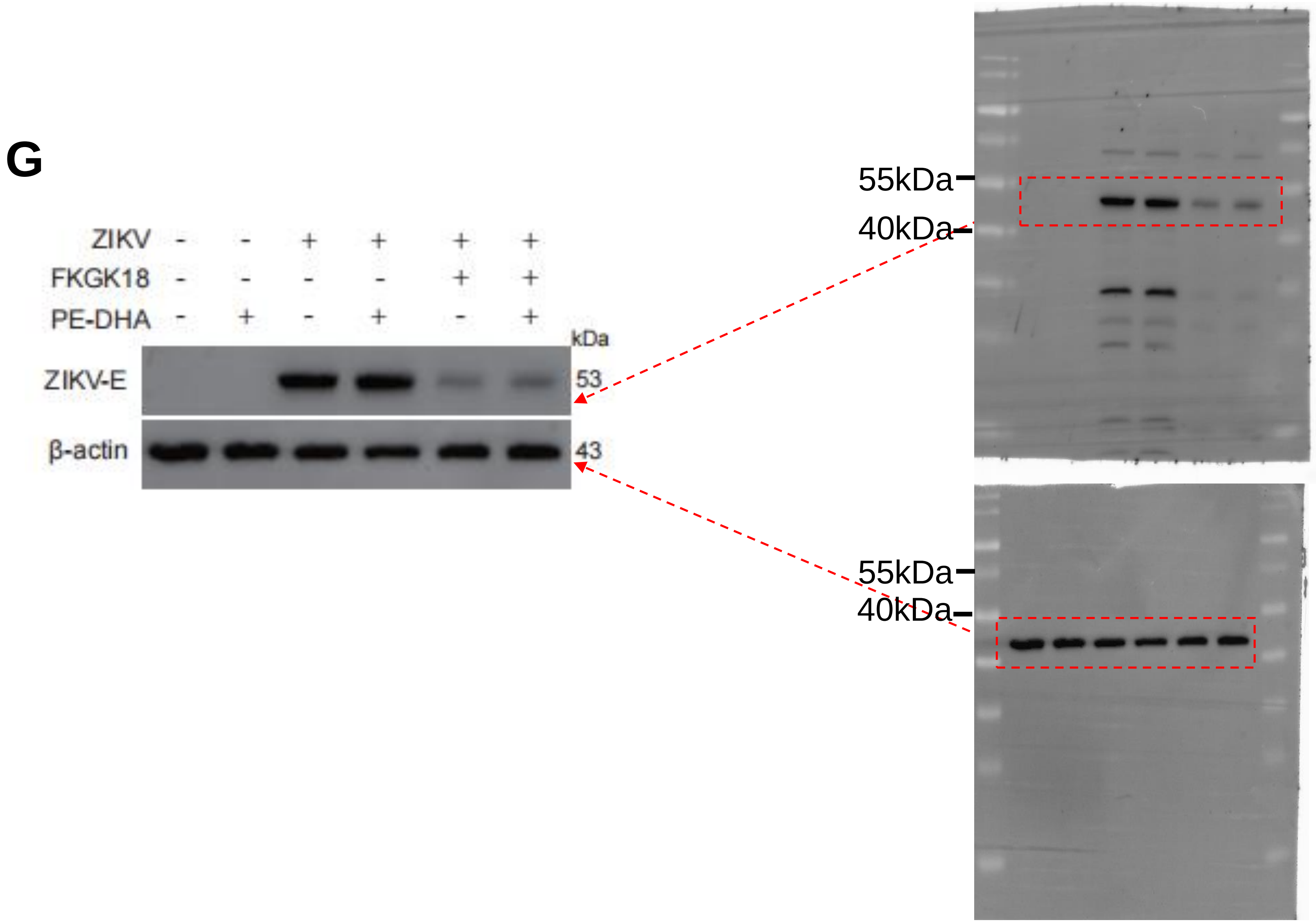
G
ZIKV - - + + + +
FKGK18 - - - - + +
PE-DHA - + - + - +
kDa
ZIKV-E
53
β-actin
43
55kDa
40kDa
55kDa
40kDa

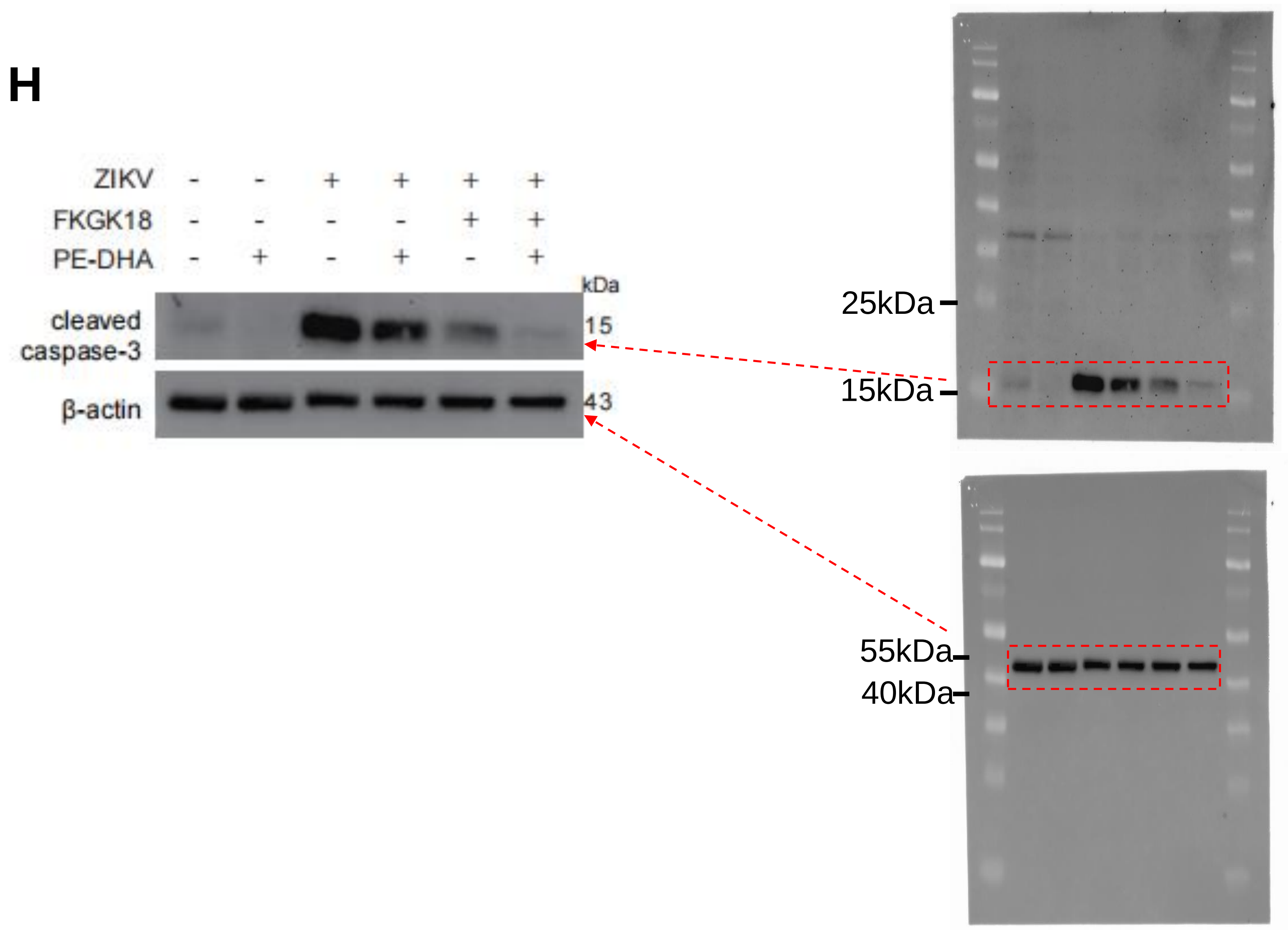
H
ZIKV - - + + + +
FKGK18 - - - - + +
PE-DHA - + - + - +
kDa
cleaved caspase-3
15
β-actin
43
25kDa
15kDa
55kDa
40kDa

Fig S2

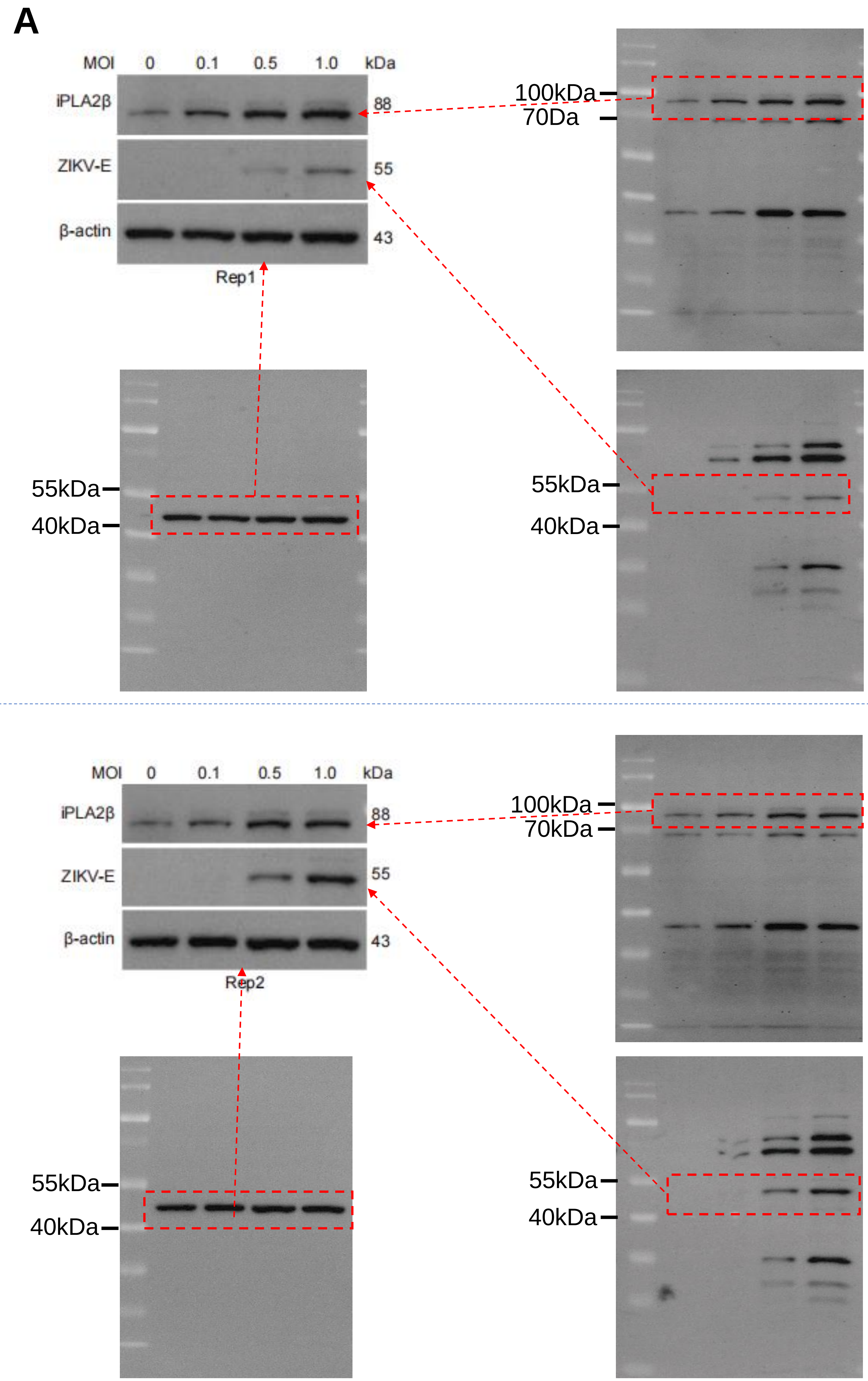
A
MOI
0
0.1
0.5
1.0
kDa
iPLA2β
88
ZIKV-E
55
β-actin
43
Rep1
100kDa
70Da
55kDa
40kDa
55kDa
40kDa
MOI
0
0.1
0.5
1.0
kDa
iPLA2β
88
ZIKV-E
55
β-actin
43
Rep2
100kDa
70kDa
55kDa
40kDa
55kDa
40kDa

Fig S2

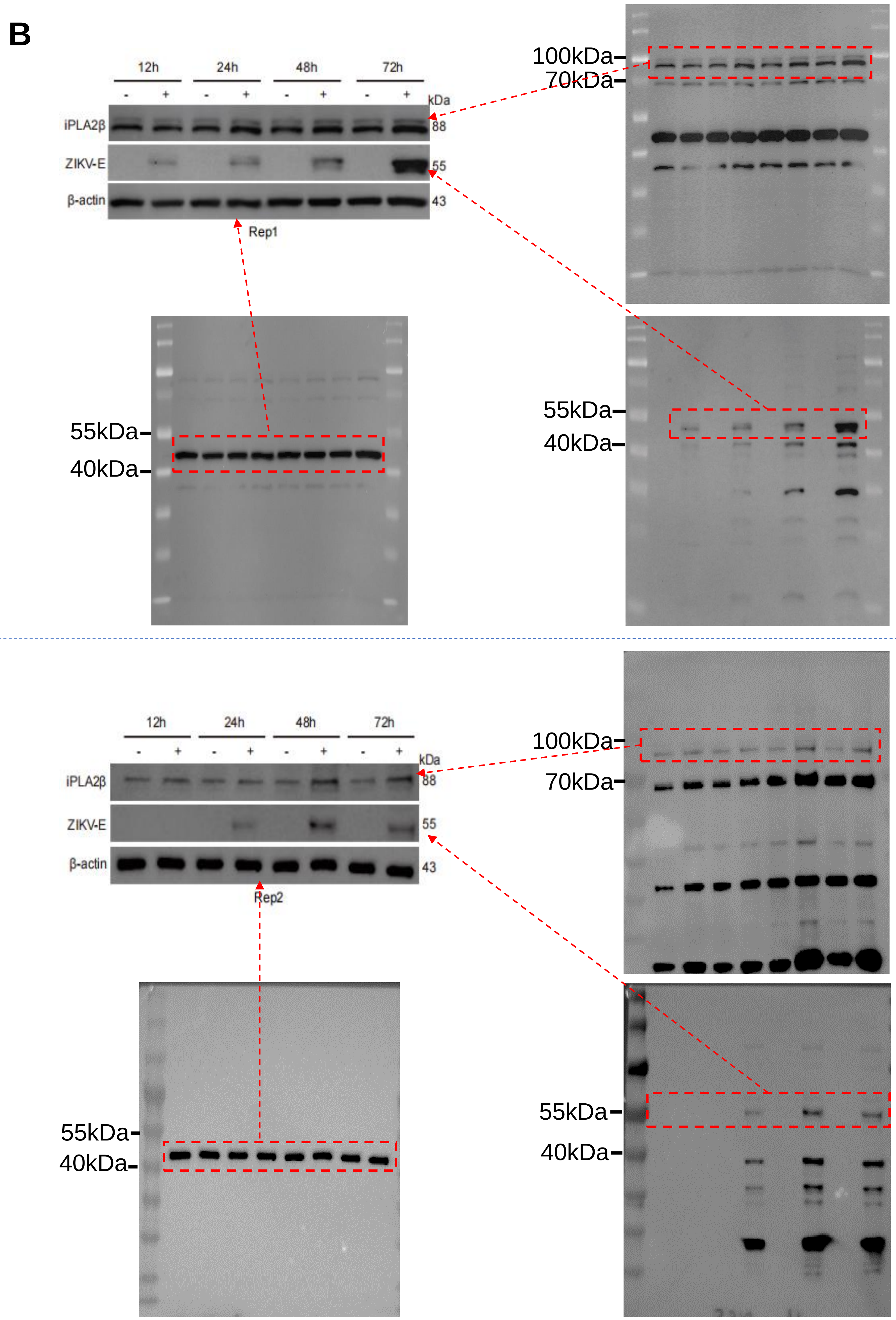
B
12h
24h
48h
72h
-
+
kDa
iPLA2β
ZIKV-E
β-actin
88
55
43
Rep1
100kDa
70kDa
55kDa
40kDa
Rep2

Fig S4

C

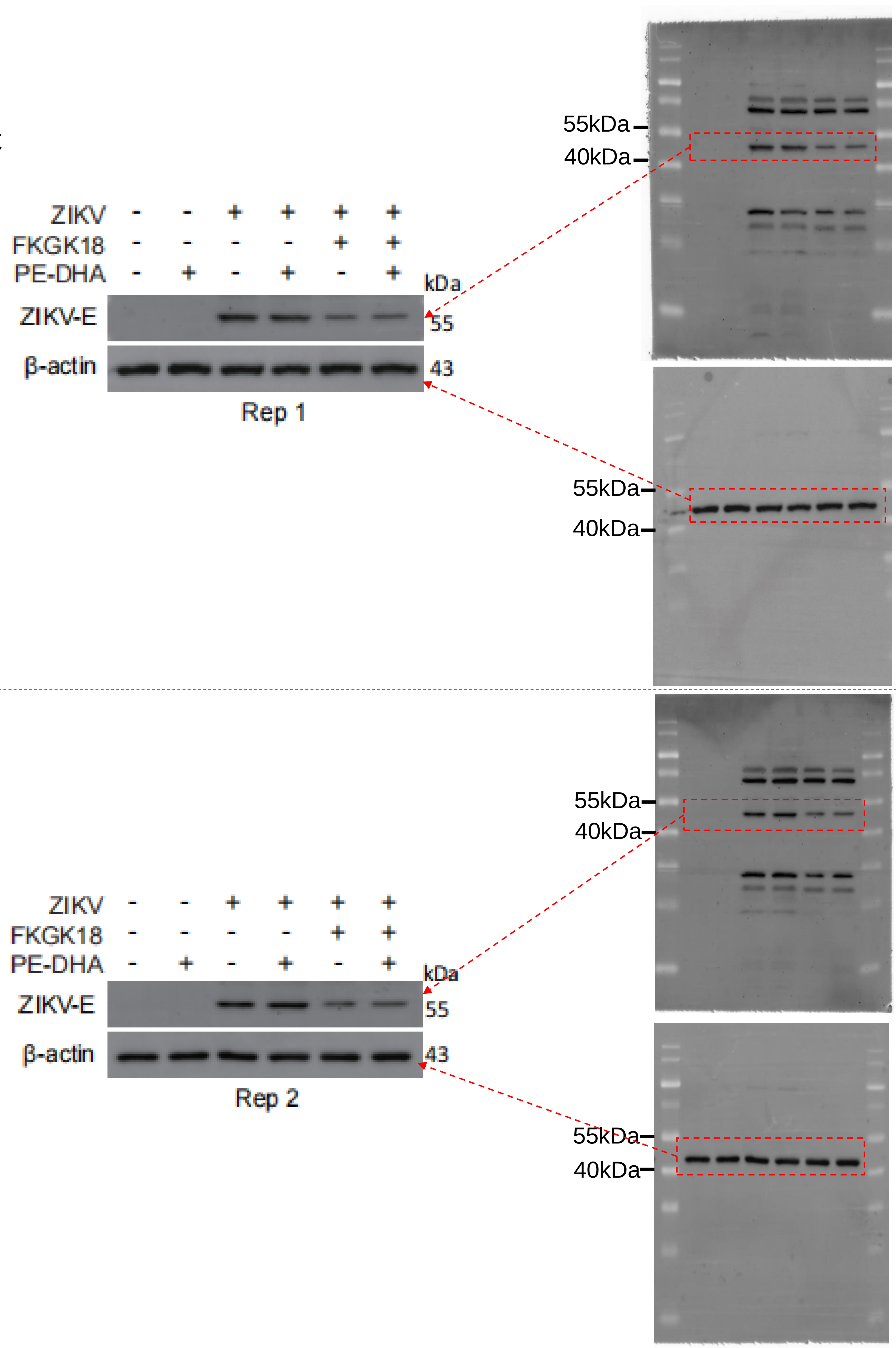

ZIKV - - + + + +
FKGK18 - - - - + +
PE-DHA - + - + - +
kDa
ZIKV-E
55
β-actin
43
Rep 1
55kDa
40kDa
55kDa
40kDa
ZIKV - - + + + +
FKGK18 - - - - + +
PE-DHA - + - + - +
kDa
ZIKV-E
55
β-actin
43
Rep 2
55kDa
40kDa
55kDa
40kDa

Fig S4

D

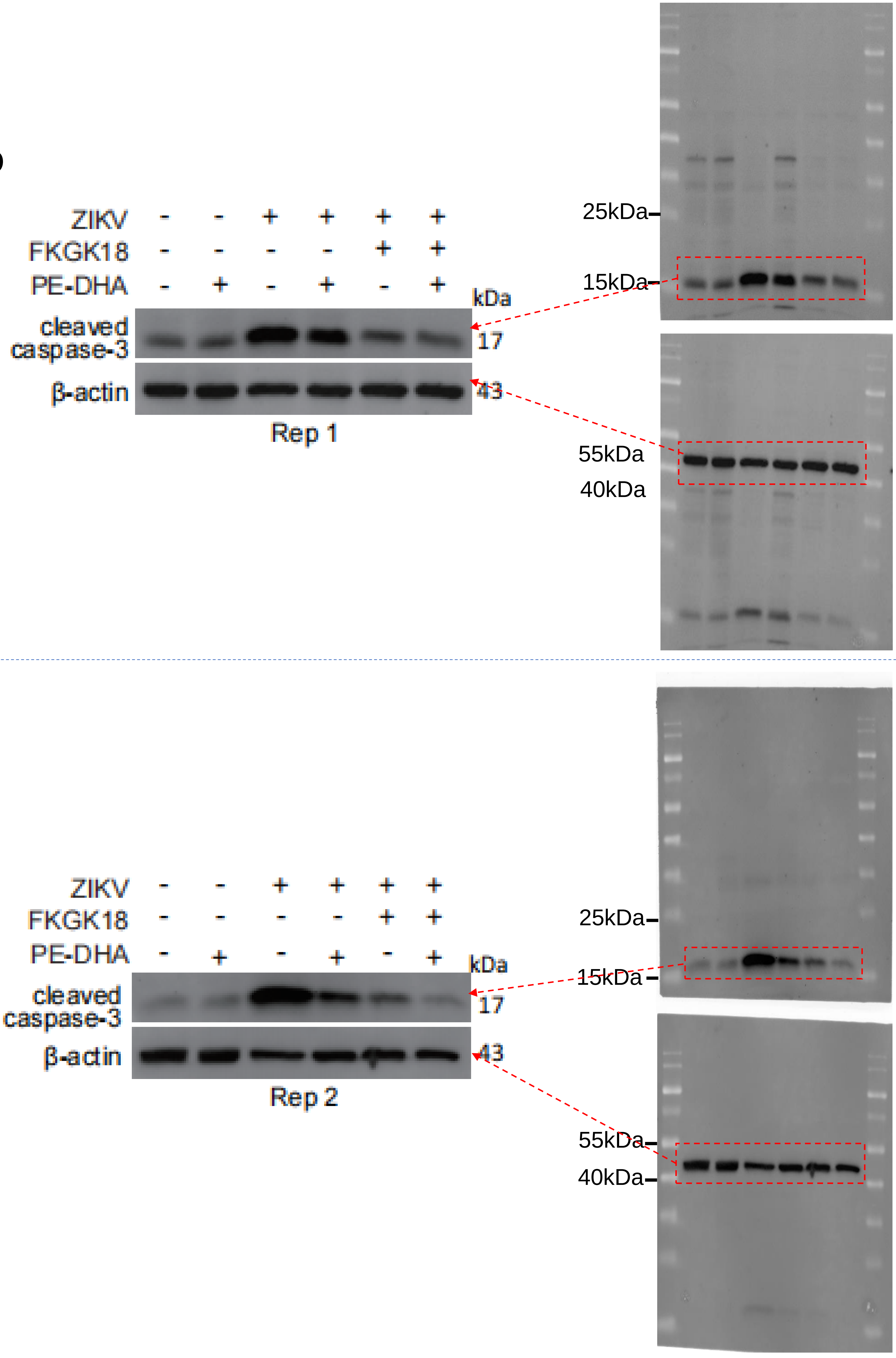
ZIKV - - + + + +
FKGK18 - - - - + +
PE-DHA - + - + - +
kDa
cleaved caspase-3
17
β-actin
43
Rep 1
25kDa
15kDa
55kDa
40kDa
ZIKV - - + + + +
FKGK18 - - - - + +
PE-DHA - + - + - +
kDa
cleaved caspase-3
17
β-actin
43
Rep 2
25kDa
15kDa
55kDa
40kDa
